# Identification and Analysis of the *GASR* Gene Family in Common Wheat (*Triticum aestivum* L.) and Characterization of *TaGASR34*, a Gene Associated With Seed Dormancy and Germination

**DOI:** 10.3389/fgene.2019.00980

**Published:** 2019-10-18

**Authors:** Xinran Cheng, Shengxing Wang, Dongmei Xu, Xue Liu, Xinyu Li, Weiwei Xiao, Jiajia Cao, Hao Jiang, Xiaoyu Min, Jianfeng Wang, Haiping Zhang, Cheng Chang, Jie Lu, Chuanxi Ma

**Affiliations:** College of Agronomy, Anhui Agricultural University, Key Laboratory of Wheat Biology and Genetic Improvement on Southern Yellow & Huai River Valley, Ministry of Agriculture and Rural Affairs, Hefei, China

**Keywords:** common wheat, seed dormancy, *GASR*, GA, ABA

## Abstract

Seed dormancy and germination are important agronomic traits in wheat (*Triticum aestivum* L.) because they determine pre-harvest sprouting (PHS) resistance and thus affect grain production. These processes are regulated by *Gibberellic Acid-Stimulated Regulator* (*GASR*) genes. In this study, we identified 37 *GASR* genes in common wheat, which were designated *TaGASR1-37*. Moreover, we identified 40 pairs of paralogous genes, of which only one had a Ka/Ks value greater than 1, indicating that most *TaGASR* genes have undergone negative selection. Chromosomal location and duplication analysis revealed 25 pairs of segmentally duplicated genes and seven pairs of tandemly duplicated genes, suggesting that large-scale duplication events may have contributed to the expansion of *TaGASR* gene family. Microarray analysis of the expression of 18 *TaGASR* genes indicated that these genes play diverse roles in different biological processes. Using wheat varieties with contrasting seed dormancy phenotypes, we investigated the expression patterns of *TaGASR* genes and the corresponding seed germination index phenotypes in response to water imbibition, exogenous ABA and GA treatment, and low- and high-temperature treatment. Based on these data, we identified the *TaGASR34* gene as potentially associated with seed dormancy and germination. Further, we used a SNP mutation of the *TaGASR34* promoter (-16) to develop the CAPS marker GS34-7B, which was then used to validate the association of *TaGASR34* with seed dormancy and germination by evaluating two natural populations across environments. Notably, the frequency of the high-dormancy *GS34-7Bb* allele was significantly lower than that of the low-dormancy *GS34-7Ba* allele, implying that the favorable *GS34-7Bb* allele has not previously been used in wheat breeding. These results provide valuable information for further functional analysis of *TaGASR* genes and present a useful gene and marker combination for future improvement of PHS resistance in wheat.

## Introduction

Common wheat (*Triticum aestivum* L.) is an important food crop grown throughout the world. One of the most important agronomic traits for wheat production is seed dormancy, which is defined as the prevention of germination of an intact viable seed under favorable conditions (Bewley, 1997). In modern varieties of domesticated wheat, low levels of dormancy (or lack of dormancy) have been selected to achieve higher yield by fast and uniform germination of seeds. However, this strategy has undesirable side effects as, under conditions of excess rainfall or humidity during harvest, low dormancy may promote germination of mature seeds while they remain within the spike of the mother plant (a phenomenon known as pre-harvest sprouting, PHS) (Clerkx et al., 2003; Finkelstein et al., 2008). It is estimated that global direct losses caused by PHS amount to one billion USD annually (Brown et al., 2018). Therefore, improving our understanding of the molecular mechanisms involved in seed dormancy and germination may be helpful for the improvement of PHS resistance in cultivated wheat.

Abscisic acid (ABA) and gibberellic acid (GA, also known as gibberellin) are two plant hormones that have decisive roles in regulating seed dormancy and germination. ABA is involved in the induction and maintenance of dormancy, whereas GA regulates the breaking of seed dormancy and thereby promotes germination (Kucera et al., 2005; Finkelstein et al., 2008). The roles of ABA and GA in dormancy and germination have been conﬁrmed by physiological, biochemical, and genetic evidence in diverse plant species (Koornneef and van der Veen, 1980; Koornneef et al., 1984; Jacobsen and Olszewski, 1993; Finkelstein et al., 2002; Lee et al., 2002; Nambara and Marion-Poll, 2003; Kushiro et al., 2004; Appleford et al., 2007; Yamauchi et al., 2007; Shu et al., 2013; Ibrahim, 2016; Huang et al., 2016; Shu et al., 2017). For example, the tobacco ABA biosynthesis gene encoding 9-cis-epoxycarotenoid dioxygenase (*LeNCED1*) has been shown to enhance seed dormancy when overexpressed (Thompson et al., 2000). In *Arabidopsis thaliana*, three mutations in *ABA-insensitive 1*, *-2*, and *-3* genes, known as *abi1*, *abi2*, and *abi3*, respectively, are associated with reduced seed dormancy (Koornneef et al., 1984). In addition, overexpression of the runner bean GA catabolism gene *GA2-oxidase 1* (*PcGA2ox1*) has been shown to be associated with increased seed dormancy in transgenic wheat (Appleford et al., 2007). GA-deﬁcient mutants (including *ga1* and *ga2*) have been found to show strong seed dormancy, since seeds of these lines did not germinate without the addition of exogenous GA (Koornneef and van der Veen, 1980; Lee et al., 2002; Yamauchi et al., 2007; Shu et al., 2013). Finally, mutations in DELLA genes such as *RGL2* (*RGA-LIKE2*) and *SPY* (*SPINDLY*), both of which are negative regulators of GA signaling, can rescue the *ga1* non-germinating seed phenotype (Jacobsen and Olszewski, 1993; Lee et al., 2002). Taken together, these findings indicate that GA and ABA synthesis and signaling are necessary to control seed dormancy and germination. However, to date the detailed mechanisms responsible for these processes, especially in hexaploid wheat, remain poorly understood.

Temperature has been shown to be an important environmental factor inﬂuencing seed dormancy. Low temperatures during seed development enhance dormancy (Rodriguez et al., 2001; Chiang et al., 2011; Kendall et al., 2011; Nakamura et al., 2011; He et al., 2014), whereas dormancy of imbibed seeds can be lost after a short exposure to low temperature (Finch-Savage and Leubner-Metzger, 2006). By contrast, incubation at high temperatures can increase the level of dormancy by affecting GA synthesis and response pathways as well as responsiveness to ABA (Walker-Simmons, 1987; Corbineau et al., 1991; Yamauchi et al., 2004; Benech-Arnold et al., 2006; Leymarie et al., 2008). These results imply the presence of crosstalk between GA and ABA synthesis and response and temperature in controlling seed dormancy and germination.

*Gibberellic Acid-Stimulated Regulator* (*GASR*, also known as *GASA* and *GAST*) genes are a family of GA-responsive genes that play important roles in regulating seed germination. GASR proteins encoded by *GASR* genes are composed of a spliceable hydrophobic signal peptide at the N-terminal, a hydrophilic region of different lengths in the middle (usually consisting of polar amino acid residues), and a C-terminal containing 12 conserved cysteines (i.e. the GASA domain) (Herzog et al., 1995; Ben-Nissan et al., 2004; De la Fuente et al., 2006; Tomoyuki et al., 2006; Zimmermann et al., 2010; Ling et al., 2013). Bioinformatic analysis has identified a GA response element (GARE), an ABA response element (ABRE), and other GA- and ABA-related cis-elements in the *GASA* promoter (Zhang and Wang, 2008), indicating a relationship between *GASA* genes and these two plant hormones.

Since Shi et al. (1992) first identified a *GAST* gene (*GAST1*) in tomato (*Solanum lycopersicon*), numerous *GAST1* homologues have subsequently been identified in other plant species, including *At*GASA in *Arabidopsis* (Herzog et al., 1995; Aubert et al., 1998), *OsGASR* in rice (*Oryza sativa*) (Furukawa et al., 2006), *StSN* in potato (*Solanum tuberosum*) (Segura et al., 1999; Berrocal-Lobo et al., 2002), ZmGSL in maize (*Zea mays*) (Zimmermann et al., 2010), *GEG* in gerbera (*Gerbera hybrida*) (Kotilainen, 1999), FaGAST in strawberry (*Fragaria vesca*) (De la Fuente et al., 2006; Moyano-Cañete et al., 2013), MdGASA in apple (Malus domestica) (Fan et al., 2017), and *GIP* in Petunia (*Petunia hybrida*) (Ben-Nissan and Weiss, 1996; Ben-Nissan et al., 2004).

Members of the GASR family are involved in diverse plant growth, development, and biotic/abiotic stress response functions, including shoot and petal growth (Shi et al., 1992; Ben-Nissan and Weiss, 1996), stem growth (Ben-Nissan et al., 2004; Wigoda et al., 2006; Zhang et al., 2009), leaf expansion (Sun et al., 2013), root formation (Taylor and Scheuring, 1994; Zimmermann et al., 2010), ﬂowering time regulation (Herzog et al., 1995; Zhang et al., 2009), seed growth and maturation (Roxrud et al., 2007; Dong et al., 2014; Zhang et al., 2016; Li et al., 2017), seed germination (Rubinovich and Weiss, 2010), fruit development and ripening (De la Fuente et al., 2006; Moyano-Cañete et al., 2013), ﬁber development (Liu et al., 2013), and heat tolerance (Ko et al., 2007; Zhang and Wang, 2011), as well as plant responses to saline (Alonso-Ramirez et al., 2009), oxidative (Wigoda et al., 2006; Alonso-Ramirez et al., 2009), wounding, and pathogen infection stresses (Segura et al., 1999; Berrocal-Lobo et al., 2002). In addition, Rubinovich and Weiss (2010) reported that seeds overexpressing *GASA4* showed partial resistance to paclobutrazol (an inhibitor of GA biosynthesis) and a higher germination percentage than wild-type *Arabidopsis* seeds. The same study also reported higher rates of germination in seeds containing artificial miR^GASA^ RNA to suppress *GASA5*, a repressor of the GA response. Similarly, Alonso-Ramirez et al. (2009) reported that overexpressing *FsGASA4*, a GASA-family gene found in *Fagus sylvatica*, increased the seed germination rates of transgenic *Arabidopsis* exposed to saline, oxidative, and heat stress. Moreover, Zhang and Wang (2008) reported that *GASA4* expression was induced by GA_3_ and inhibited by ABA, whereas *GASA5* expression showed the opposite trend. With respect to *GASA6*, Zhong et al. (2015) reported that *AtGASA6*-overexpressing seeds displayed early germination, whereas reduced *AtGASA6* expression in transfer DNA (T-DNA) insertion and RNA interference (RNAi) knockout/knockdown mutants resulted in delayed seed germination in response to ABA, paclobutrazol, and glucose (Glc) stress treatments. These results suggest that *AtGASA6* integrates GA, ABA, and Glc signaling in the regulation of seed germination. Taken together, *GASA4*, *GASA5*, and *GASA6* likely play an important role in controlling dormancy and germination by modulating plant responses to GA and ABA. However, the roles that these *GASA* homologs play in common wheat remain unclear.

The objectives of this study were to identify *GASR* genes in wheat (*TaGASR* genes) and perform bioinformatic analyses, including the generation of a phylogenetic tree and the examination of gene structure, conserved domains, chromosomal location, expression patterns, duplication events, and promoter sequences; clone *TaGASR* genes associated with seed dormancy and germination and introduce these *TaGASR* genes into wheat varieties with contrasting seed dormancy phenotypes; and validate the association of *TaGASR* genes with seed dormancy and germination in different natural populations.

## Materials and Methods

### Plant Materials and Field Trials

Six wheat varieties with contrasting dormancy levels were selected to examine the expression of *TaGASR* genes. These included: Zhongmai 895 (ZM895, average germination index (GI): 0.92), Jing 411 (J411, average GI: 0.96), Zhongyou 9507 (ZY9507, average GI: 0.97), Yangxiaomai (YXM, average GI: 0.07), Suiningtuotuo (SNTT, Caverage GI: 0.15), Hongmangchun 21 (HMC21, average GI: 0.09). Varieties J411 and HMC21, which have especially low and especially high levels of seed dormancy, respectively, were used for cloning the *TaGASR34* gene.

We validated the association of *TaGASR34* with seed dormancy and germination using the Chinese wheat mini-core collection (CMCC), a small core collection consisting of 260 Chinese wheat varieties (**Table S1**), as well as a natural population (NP) consisting of 260 Chinese wheat varieties (including 179 from the Yellow and Huai Valleys Winter Wheat region, 30 from the Southwest Winter Wheat region, 22 from the Middle and Lower Yangtze River Valley Winter Wheat region, 21 from the Northern Winter Wheat region, and 8 from outside China) (**Table S2**). The CMCC and NP were planted at the Dayangdian experimental station of Anhui Agricultural University in Hefei, China (31° 58′ N, 117° 240′ E). CMCC plants were grown during the 2014–2015 and 2015–2016 growing seasons, and NP plants were grown during the 2013–2014 and 2014–2015 growing seasons. Field trials were conducted in plots containing two 2 m rows 25 cm apart. Forty seeds were planted in each row. All experiments were performed in randomized complete blocks with two independent replicates. Field management followed local agricultural practices.

Flowering time was scored when 50% of florets were open in a plot. Sixty spikes of each plot were collected at physiological maturity (i.e. after loss of chlorophyll from the spike, leaf and peduncle) (Trethowan, 1995), naturally air dried for 3 days avoiding direct sunlight and high temperature, hand-threshed to minimize damage to embryos and seed coat, then stored at -20°C until all were harvested. After all varieties were threshed, they were used for subsequent seed germination index (GI) assay.

### Germination Index Assays

Fifty seeds from each genotype were placed in Φ 90 Petri dishes on ﬁlter paper with 9 ml distilled water, and then grown in a 20°C greenhouse with a 14 h day/10 h night photoperiod cycle at 80% humidity. The number of germinated seeds in each culture dish was counted at the same time every day and removed. The GI values were calculated after 7 days. Germination was defined as visible rupture of the pericarp and testa (Mares, 1983; Chang et al., 2010).

All GI tests were conducted twice at 5 and 15 days after harvest. For CMCC plants, GI measurements made 5 and 15 days after harvest in 2014, 2015, and 2016 were designated 14GI5-CMCC, 14GI15-CMCC, 15GI5-CMCC, 15GI15-CMCC, 16GI5-CMCC, and 16GI15-CMCC, respectively. For NP plants, GI measurements made 5 and 15 after harvest in 2013, 2014, and 2015 were designated 13GI5-NP, 13GI15-NP, 14GI5-NP, 14GI15-NP, 15GI5-NP, and 15GI15-NP, respectively.

### Identification of *TaGASR* Genes in Common Wheat

We obtained the full sequence of the wheat genome from the Ensembl database (http://plants.ensembl.org/index.html). All candidate *TaGASR* gene sequences were obtained by BLAST search using a hidden Markov model (HMM) of the Pfam database. Sequences of candidate genes were confirmed by querying the Pfam, SMART, and NCBI databases (Chen et al., 2015). Bioinformatics analysis of the *TaGASR* genes were performed, including the determination of ORFs and the calculation of pI values, Mw values, and nucleic acid lengths of all genes, using the ExPASy website (www.expasy.org).

### Phylogenetic Tree, Multiple Alignment and Gene Structure Analysis

Phylogenetic trees were constructed using the NJ method as implemented by MEGA version 7 with the number of bootstraps set to 1,000 (Chu et al., 2016; Cheng et al., 2018). In addition, the CDS and gene sequences of *TaGASR* genes were analyzed using Gene Structure Display Server (GSDS) version 2.0 (gsds.cbi.pku.edu.cn) to determine the structure of their exons/introns (Wang et al., 2017; Cheng et al., 2018). Multiple sequence alignments of the 37 *TaGASR* full-length protein sequences were performed using ClustalX 2.11 (Gao et al., 2017; Liu et al., 2017).

### Conserved Domain and Promoter Analysis

We used MEME Suite version 5.0.5 to identify conservative motifs, and performed all searches using the default parameter settings (Wu et al., 2016). We also used the PlantCARE database (http://bioinformatics.psb.ugent.be/webtools/plantcare/html) to analyze the regions 1,500 bp up- and downstream of *TaGASR* family genes in order to identify the type and number of cis-acting elements in the promoters of these genes (Zhao et al., 2018).

### Microarray Analysis

We obtained microarray data for three biological replicates of 13 different tissue samples from the Gene Expression Omnibus (https://www.ncbi.nlm.nih.gov/geo/) database of the National Center for Biotechnology Information (NCBI) (Barrett and Edgar, 2006) using the login number GSE12508. The online probe matching tool provided by the NetAffx Analysis Center (Wilkins et al., 2009) (https://www.affymetrix.com/analysis/index.affx) was used to identify the probes corresponding to the putative *TaGASR* genes. When the gene had more than one probe group, the probe with the highest matching value was used. All data was normalized, logarithmized, averaged, and saved as tab-delimited files before importing into Cluster (version 3.0) (Sturn et al., 2002) to generate heat maps. Finally, heat maps were obtained using Heat-mapper Plus (www.heatmapper.ca) (Kiana et al., 2005).

### Identifying Homologous Pairs and Calculating Ka/Ks Values

Paralogous pairs (gene pairs originating from duplication events within genome of a single species) and orthologous pairs (gene pairs in different genomes that have diverged by speciation) were identified according to the method described in Altschul et al. (1997). We identified paralogous pairs as aligned sequences longer than 300 bp with identity ≥ 40%, and identified orthologous pairs as aligned sequences longer than 300 bp (Blanc and Wolfe, 2004).

Ka and Ks were calculated according to the method described in Wang et al. (2015). Sequence alignment was performed using MEGA 7.0, and Ka/Ks values were calculated using DnaSP version 5 (Wang et al., 2017).

### Chromosomal Location and Duplication Analysis

The physical locations of *TaGASR* genes were obtained from the Ensembl database and constructed chromosomal maps using MapGene2Chromosome version 2.0 (http://mg2c.iask.in/mg2c_v2.0/) (Voorrips, 2002). To classify the expansion of *TaGASR* genes, putative tandem duplications of gene family members were examined in the same gene region and in adjacent gene regions (Cannon et al., 2004). All *GASR* genes were analyzed and compared using pairwise BLASTP with E-values < 10^-10^. The coordinates of segmental duplications of target genes were searched by querying the Vista Synteny browser (pipeline.lbl.gov/cgibin/gateway2). If genes of interest were located in duplicated chromosomal blocks, these paralogs were deemed to be generated by segmental duplication. Two genes found within a 100-kb region that were separated by five or fewer genes were deemed to be tandemly duplicated. Using the Smith-Waterman algorithm (http://www.ebi.ac.uk/Tools/psa/) we calculated the local alignment between the two protein sequences of duplicated genes. Finally, we generated synchronized maps using Circos version 0.69 (Jorge et al., 2000); putative duplicated genes are connected by colored lines.

### ABA, GA, Low and High Temperature Treatments

Seeds of two wheat varieties (J411 and HMC21) were treated with 50 μM GA_3_, 50 μM ABA, low temperature (4°C), or high temperature (36°C) treatments. Distilled water was used as a control. Seed samples were collected at 48 h after the start of the treatment. Collected seeds were immediately frozen in liquid nitrogen and stored at -80°C for RNA isolation.

### Determination and Analysis of Endogenous Hormones ABA and GA

Collected seeds treated with GA_3_, ABA, low temperature, or high temperature treatments were immediately frozen in liquid nitrogen, ground into a powder, and 0.1 g of the sample was mixed with a methanol–water (80:20 V/V) solution. The standard compounds within the mixture were separated by electrospray ionization liquid chromatography tandem mass spectrometry (LC-ESI-MS/MS), as described by Yoshimoto et al. (2009). Hormones were extracted from at least three independent samples harvested.

### RNA Extraction and qRT-PCR Analysis

Total RNA was extracted from seeds by the Trizol method. cDNA was synthesized using a Primer Script RT Master Mix (Takara, Tokyo, Japan) according to the manufacturer’s instructions. Specific primers for 37 *TaGASR* genes were designed using Primer Premier 5.0 (**Table S3**), and *TaActin* was used as a reference gene (Sun et al., 2015).

The total volume of PCR reactions used for qRT-PCR analysis was 20 μl. Each reaction included 10 μl TransStart Tip Green qPCR SuperMix, 0.4 μl Passive Reference Dye, 0.4 μl each of forward and reverse primers, and 8.8 μl ddH_2_O. The reaction procedure was as follows: an initial denaturation at 94°C for 30 s, followed by 40–45 cycles of 94°C for 5 s and 50–60°C for 15 s and a final extension step at 72°C for 10 s. We performed three biological replicates for each sample. Finally, we used GraphPad version 5 to process data and generate charts (Bryfczynski, 2009).

### DNA Extraction and Cloning of *TaGASR34*

Genomic DNA was isolated from undamaged dry kernels of the J411 and HMC21 varieties using a modified phenol–chloroform method (Hu et al., 2016; Jiang et al., 2018). The full-length sequence of the *TaGASR34* gene was obtained by querying the Chinese spring wheat genome. Gene-specific primers were designed to selectively amplify the *GASR34* gene (**Table S4**). We then isolated the *GASR* gene sequence from both the J411 and HMC21 varieties. These amplicons were then cloned and sequenced (**Table S5**).

The total reaction volume used for the cloning PCR was 20 μl, including 4.0 μl TransStart^®^ FastPfu buffer, 1.6 μl 2.5 mmol/L dNTPs, 0.4 μl 2.5 U/μl TransStart^®^ FastPfu DNA polymerase, 0.4 μl each of 10 μmol/L forward and reverse primer, 2.0 μl of (50–60 ng/μl) template DNA, and 10.4 μl ddH_2_O. The cloning PCR reaction procedure was as follows: an initial denaturation at 94°C for 5 min, 37 cycles of 95°C for 30 s and 60°C for 30 s, and a final elongation at 72°C for 2 min. PCR products were then separated in 1.5% agarose gels, and the target fragment was recovered from the gel matrix. The recovered product was introduced into Trans1-T1 competent cells, gently mixed, and cultured 8 h. Liquid samples containing positive clones were identified by sequencing (Sangon Biotech, Shanghai, China).

DNAMAN version 7.0 was used to compare sequencing results to identify different allelic variations. Known sequence information from the *TaGASR34* CDS was used to analyze gene structure (e.g. promoter, exon, intron, and 3’UTR regions) and SNP variation (**Table S5**).

### Development of Gene-Specific Markers for *TaGASR34*

One gene-specific primer pair (designated GS34-7B) was designed based on a SNP mutation of the *TaGASR34* promoter (-16) using Primer Premier version 5.0 (**Table S4**). The resulting amplification product was digested by *BsaI* at 37°C to introduce one SNP mutation (C/G) in the *TaGASR34* promoter. We amplified the GS34-7B marker using the cloning PCR reaction conditions described above. The resulting PCR product was digested with *BsaI* for 6 h. Digested fragments were separated on 2% agarose gels.

### Validation of Gene-Specific Markers for *TaGASR34*

The gene-specific marker GS34-7B for *TaGASR34* was validated in the CMCC (**Table S1**) and NP groups of wheat varieties (**Table S2**). Descriptive statistics and Mann-Whitney U-tests were performed to analyze significant differences in GI values between varieties with the two alleles of GS34-7B. Our genotyping results found that the GS34-7B marker identified two alleles, including the allele *GS34-7Ba*, which was associated with higher GI values, and *GS34-7Bb*, which was associated with lower GI values.

### Statistical Analysis

Excel and SPSS version 18.0 were used for data analysis. We calculated mean values and standard deviation (SD) from three technical replicates each of three biological replicates. Student t-tests were used to determine whether there were significant differences between the mean values of treatment and control plants. The significance threshold used was *P < 0.05.

## Results

### Identification and Evolutionary Analysis of *TaGASR* Genes

We identified 37 *GASR* genes in common wheat based on a typical *GASR* motif (PF02704, one HMM model). These were designated *TaGASR1*-*37* according to the name of the species and their chromosomal location (**Table 1**). The amino acid (aa) lengths of all 37 *TaGASR* genes ranged from 261 to 1,172 aa. The longest gene was *TaGASR31*, the shortest was *TaGASR3*, and the lengths of their open reading frames (ORFs) ranged from 786–3,519 bp. The predicted protein molecular weights (MW) of TaGASR proteins ranged from 21,296.94 to 98,433.61 Da, and their theoretical isoelectric points (pI) varied between 4.99 and 5.27.

**Table 1:** Detailed information regarding putative *TaGASR* genes.

Name	Gene ID	Location	ORF length (bp)	Size (aa)	MW (Da)	pI	Exons
*TaGASR1*	TraesCS1A01G270100	1A:464115695—464116192	840	279	23,145.86	5.22	3
*TaGASR2*	TraesCS1A01G381600	1A:552181328—552182083	939	312	25,927.23	5.26	4
*TaGASR3*	TraesCS1A01G381700	1A:552201760—552202378	786	261	21,296.94	5.11	3
*TaGASR4*	TraesCS1B01G404100	1B:633320259—633321055	939	312	25,925.26	5.26	4
*TaGASR5*	TraesCS1D01G270100	1D:365244969—365245880	2,115	704	58,991.41	5.09	3
*TaGASR6*	TraesCS1D01G270300	1D:365298086—365298572	840	279	23,081.74	5.22	3
*TaGASR7*	TraesCS1D01G389400	1D:461116915—461117683	939	312	25,951.22	5.27	4
*TaGASR8*	TraesCS2A01G007700	2A:3400905—3406542	1,263	420	35,376.66	5.17	2
*TaGASR9*	TraesCS2A01G319900	2A:547776218—547776715	975	324	26,953.18	5.2	3
*TaGASR10*	TraesCS2A01G333600	2A:566986482—566986979	975	324	27,003.33	5.19	3
*TaGASR11*	TraesCS2B01G011100	2B:5993745—5998277	957	318	26,344.1	5.23	4
*TaGASR12*	TraesCS2B01G011700	2B:6132796—6133192	840	279	23,532.12	5.24	2
*TaGASR13*	TraesCS2B01G211100	2B:194906992—194907441	957	318	26,123.91	5.21	2
*TaGASR14*	TraesCS2B01G346900	2B:493650284—493650785	975	324	26,931.22	5.19	3
*TaGASR15*	TraesCS2D01G009400	2D:5118501—5118866	840	279	23,506.16	5.24	2
*TaGASR16*	TraesCS2D01G192300	2D:136468690—136469902	3,300	1,099	91,401.43	5.03	2
*TaGASR17*	TraesCS2D01G327800	2D:420990553—420991075	975	324	26,937.33	5.19	3
*TaGASR18*	TraesCS4B01G077900	4B:74908503—74908911	894	297	23,958.3	5.23	2
*TaGASR19*	TraesCS4D01G076400	4D:50888949—50889348	894	297	24,040.45	5.22	2
*TaGASR20*	TraesCS5A01G227000	5A:442723257—442724221	1,938	645	53,515.83	5.1	4
*TaGASR21*	TraesCS5A01G398500	5A:592302499—592302890	858	285	23,600.26	5.22	2
*TaGASR22*	TraesCS5A01G398600	5A:592474249—592474653	876	291	24,235.07	5.22	2
*TaGASR23*	TraesCS5A01G398700	5A:592519870—592520318	894	297	24,749.8	5.21	2
*TaGASR24*	TraesCS5B01G225600	5B:401073468—401074124	1,020	339	27,796.7	5.22	4
*TaGASR25*	TraesCS5B01G403500	5B:580115344—580115739	858	285	23,624.25	5.22	2
*TaGASR26*	TraesCS5B01G403600	5B:580124431—580125178	1,839	612	51,150.81	5.09	2
*TaGASR27*	TraesCS5D01G234400	5D:341578621—341579336	1,011	336	27,463.45	5.21	4
*TaGASR28*	TraesCS5D01G408000	5D:472745616—472746020	858	285	23,644.32	5.22	2
*TaGASR29*	TraesCS5D01G408100	5D:472768198—472768936	1,767	588	49,414.99	5.1	2
*TaGASR30*	TraesCS6A01G413200	6A:614520832—614521783	1,191	396	33,322.77	5.15	2
*TaGASR31*	TraesCS6B01G462400	6B:715627952—715629949	3,519	1,172	98,433.61	4.99	2
*TaGASR32*	TraesCS6D01G397800	6D:469391551—469392718	1,092	363	30,428.33	5.18	2
*TaGASR33*	TraesCS7A01G208100	7A:170684473—170684995	921	306	24,944.54	5.22	3
*TaGASR34*	TraesCS7B01G115300	7B:133792267—133792787	903	300	24,634.35	5.22	3
*TaGASR35*	TraesCS7B01G484400	7B:741572804—741573353	1,110	369	31,614.46	5.14	3
*TaGASR36*	TraesCS7D01G210500	7D:168494111—168495028	2,139	712	59,399.58	5.08	3
*TaGASR37*	TraesCS7D01G550800	7D:634690772—634691367	1,173	390	33,401.22	5.14	3

**Figure 1:**
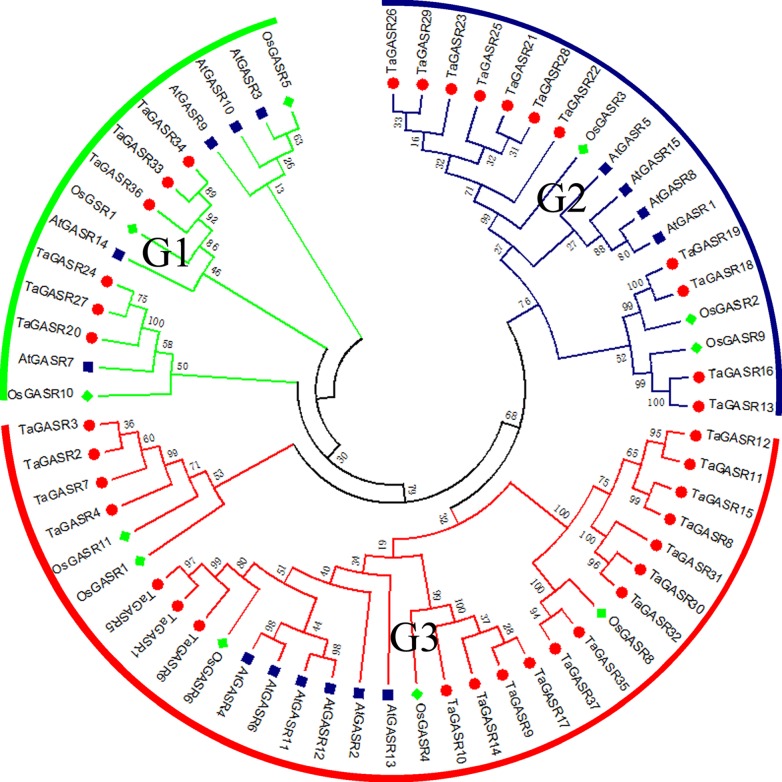
Phylogeny of GASRs from wheat, rice and Arabidopsis. The 37 *TaGASR* genes, 11 *OsGASR* genes, and 15 *AtGASR* genes are clustered into three subfamilies. Details of *GASR* genes from *Arabidopsis* and rice are listed in **Table S6**. The tree was generated using ClustalX version 2.11 using the neighbor-joining (NJ) method.

Next, we constructed a phylogenetic tree of all *GASR* family genes. Based on the classification of *GASR* genes in rice and *Arabidopsis* (**Table S6**), members of the *GASR* gene family in the phylogenetic tree were divided into three subfamilies (G1, G2, and G3) (**Figure 1**). Of these, subfamily G3 contained the most members (20), while subfamily G1 had the fewest members (6).

**Figure 2:**
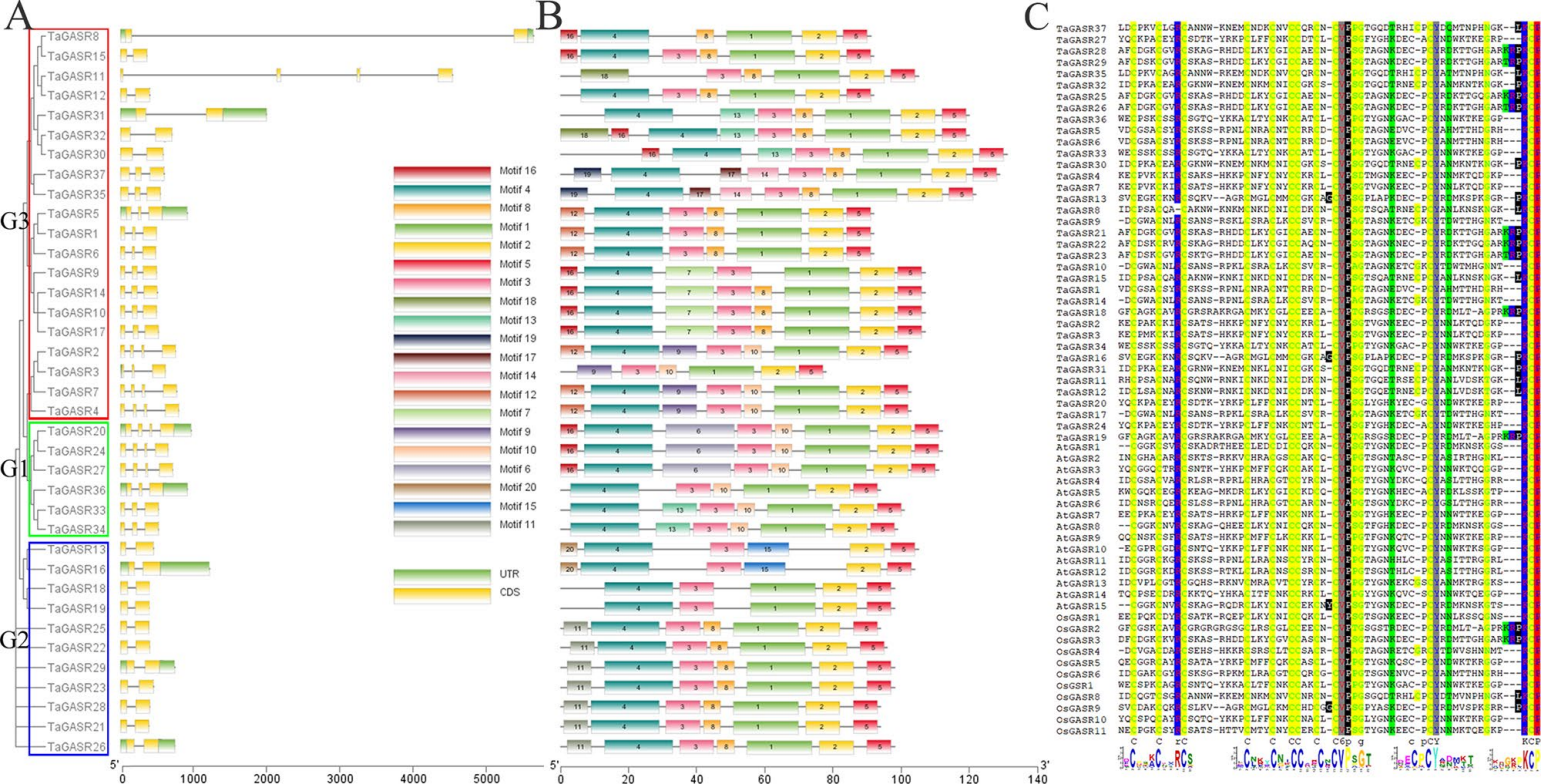
Evolutionary and gene structure analysis of TaGASR genes. **(A)** Phylogenetic relationships and gene structures of *TaGASR* genes. Exons, introns and untranslated regions (UTRs) are indicated by yellow rectangles, gray lines, and green rectangles, respectively. Colored boxes indicate the subfamily based on the phylogenetic analysis. **(B)** Schematic representation of 20 conserved motifs in *TaGASR* genes. Conserved motifs in *TaGASR* genes were identified using MEME. Different colored boxes represent different motifs. Box lengths in the figure do not represent actual relative motif sizes. **(C)** Multiple sequence alignment of TaGASR proteins. Sequences were aligned using DNAMAN software. The GASA motif is clearly highly conserved.

Most *TaGASR* genes had 2–4 exons (**Figure 2A**). In addition, 37 (92.5%) of the 40 paralogous pairs had the same number of exons and similar gene structures (**Figure 2A**). Twenty motifs were detected in the 37 *TaGASR* gene family members using MEME. Among these, motif 2 (a variable region) and motif 5 (a GASR domain) were identified in all *TaGASR* genes, and motif 3 (a putative signal peptide) was found in 36 *TaGASR* genes (all except *TaGASR8*; **Figure 2B**). In addition, multiple alignment analysis of GASR protein sequences of rice, *Arabidopsis thaliana*, and wheat showed that all putative TaGASR proteins had a conserved GASA domain (**Figure 2C**).

### Promoter and Microarray Analysis of the *TaGASR* Gene Family

**Figure 3:**
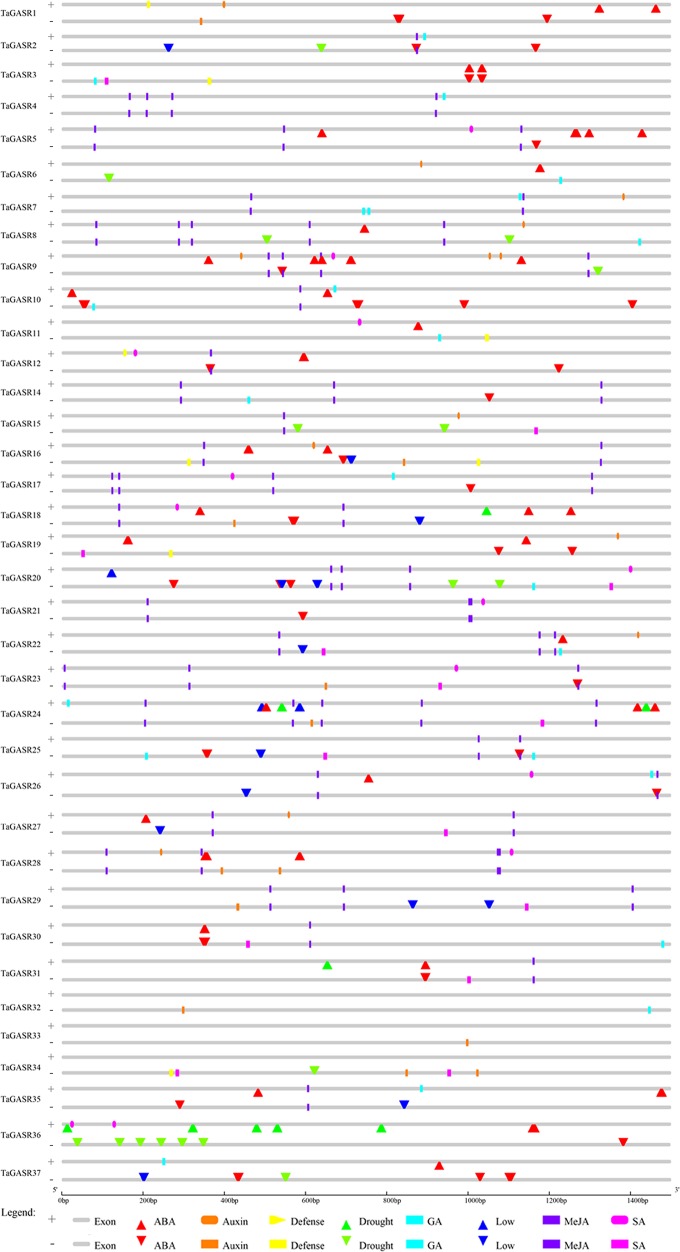
Cis-acting element analysis of the promoter regions of TaGASR genes. Based on functional annotation data, cis-acting elements were classified into two major classes: phytohormone responsive elements (i.e. those responsive to ABA, auxin, GA, MeJA, and/or SA) and abiotic stress response cis-acting elements (e.g. those involved in plant defense, drought stress response, and/or low temperature stress response).

Thirty-six *TaGASR* genes (i.e. all putative genes except *TaGASR13*) were found to contain two components. One is a cis-acting regulatory element that responsive to biological stress. These elements, include CGTCA- (present in 23.41% of all putative *TaGASR* genes) and/or TGACG-motifs (23.41%) involved in plant methyl jasmonic acid (MeJA) response, TCA-elements (9.57%) involved in salicylic acid (SA) response, ABREs (29.79%) involved in ABA response, GARE-motifs (2.48%), and P-boxes (1.77%) involved in GA response, and AuxRR-cores (1.06%) and TGA-elements (8.51%) involved in auxin (IAA) response (**Figure 3** and **Table S7**). The second element was a cis-acting regulatory element involved in abiotic stress responses, including low temperature stress response (LTR), drought inducibility (MBS), and general defense and stress response cis-acting elements (TC-rich repeats) (**Figure 3** and **Table S7**).

**Figure 4:**
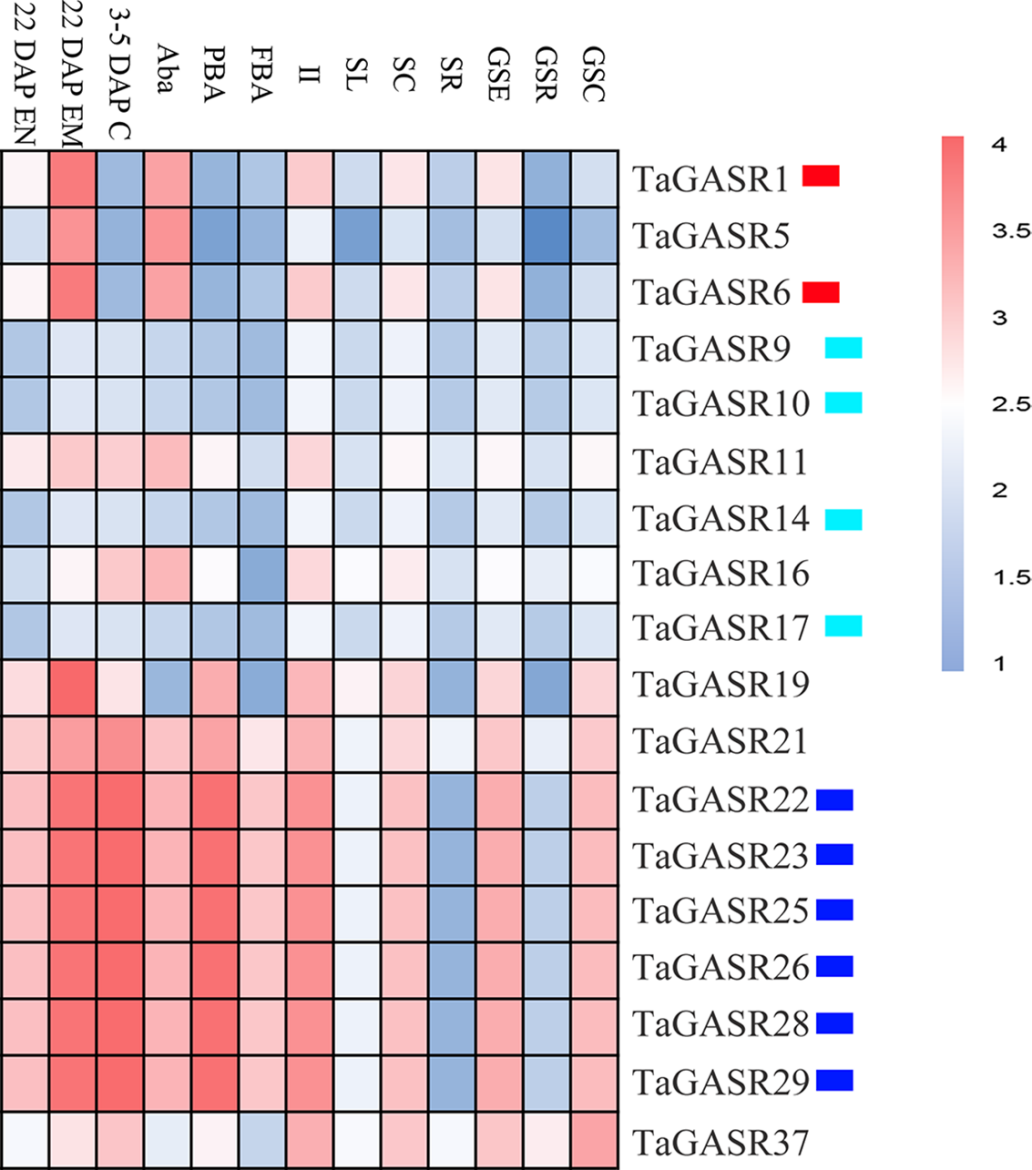
Expression profiles of TaGASR genes in different tissues and at different developmental stages. Heatmap shows hierarchical clustering of the 18 *TaGASR* genes among different tissues. Abbreviations represent specific developmental stages: GSC, germinating seed, coleoptile; GSR, germinating seed, root; GSE, germinating seed, embryo; SR, seedling, root; SC, seedling, crown; SL, seedling, leaf; II, immature inflorescence; FBA, floral bracts, before anthesis; PBA, pistil, before anthesis; Aba, anthers, before anthesis; 3–5 DAP C, 3–5 DAP caryopsis; 22 DAP EM , 22 DAP embryo; 22 DAP EN, 22 DAP endosperm.

Microarray expression data was obtained for 18 of the *TaGASR* genes from the NCBI database (accession number GSE12508). Most *TaGASR* genes showed tissue-specific expression patterns. In particular, *TaGASR1*, *TaGASR5*, and *TaGASR6* were highly expressed in anthers, both before anthesis (Aba) and 22 days post-anthesis (22.DAP.EM). Moreover, we obtained microarray data showing the relative expression levels of 27 of the 40 paralogous pairs in the *TaGASR* gene family. Of these, 22 showed similar expression patterns, and five (i.e. *TaGASR1*/-*5*, *TaGASR5*/-*6*, *TaGASR21*/-*22*, *TaGASR21*/-*25*, and *TaGASR21*/-*28*) showed differential expression patterns (**Figure 4** and **Table S8**).

### Chromosomal Location and Duplication Analysis of the *TaGASR* Gene Family

**Figure 5:**
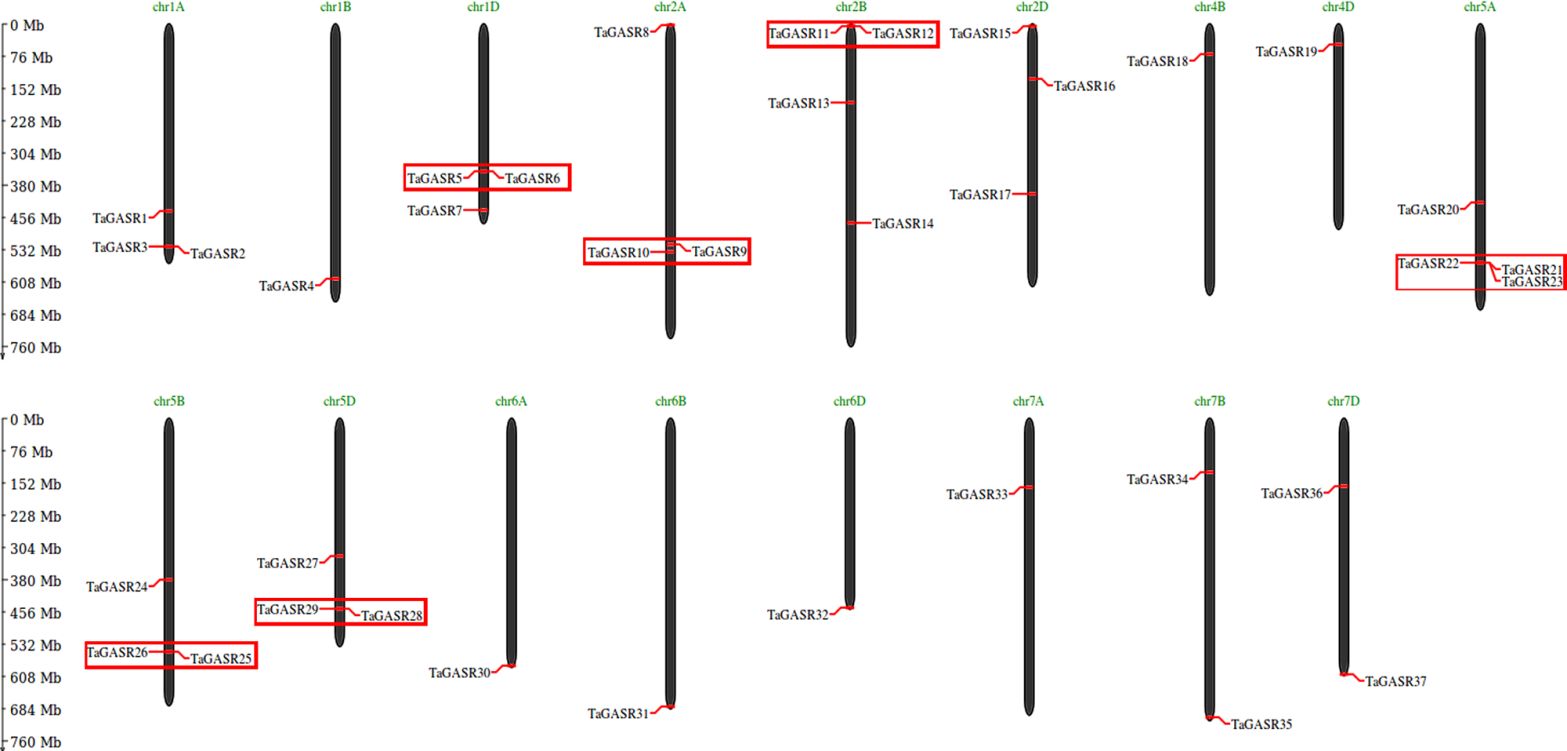
Chromosomal localization and gene duplication events of TaGASR genes. Respective chromosome numbers are indicated above each bar. Duplicated paralogous pairs of *GASR* genes in tandem duplication blocks are indicated by small boxes of the same color.

Thirty-seven *TaGASR* genes were distributed on wheat chromosome groups 1–7, except none were found on groups 3 and 4A (**Figure 5**). More than three genes each were found on chromosomes 1A, 1D, 2A, 2B, 2D, 5A, 5B, and 5D, and four were present on chromosomes 2B and 5A. Other chromosomes contained fewer than three *TaGASR* genes. According to Sturn et al. (2002), chromosomal regions smaller than 200 Kb containing two or more genes can be defined as a single gene cluster. In this study, we identified six gene clusters containing a total of thirteen genes of the *TaGASR* gene family. These were evenly distributed on chromosomes 1D, 2A, 2B, 5A, 5B, and 5D (**Figure 5**).

**Figure 6:**
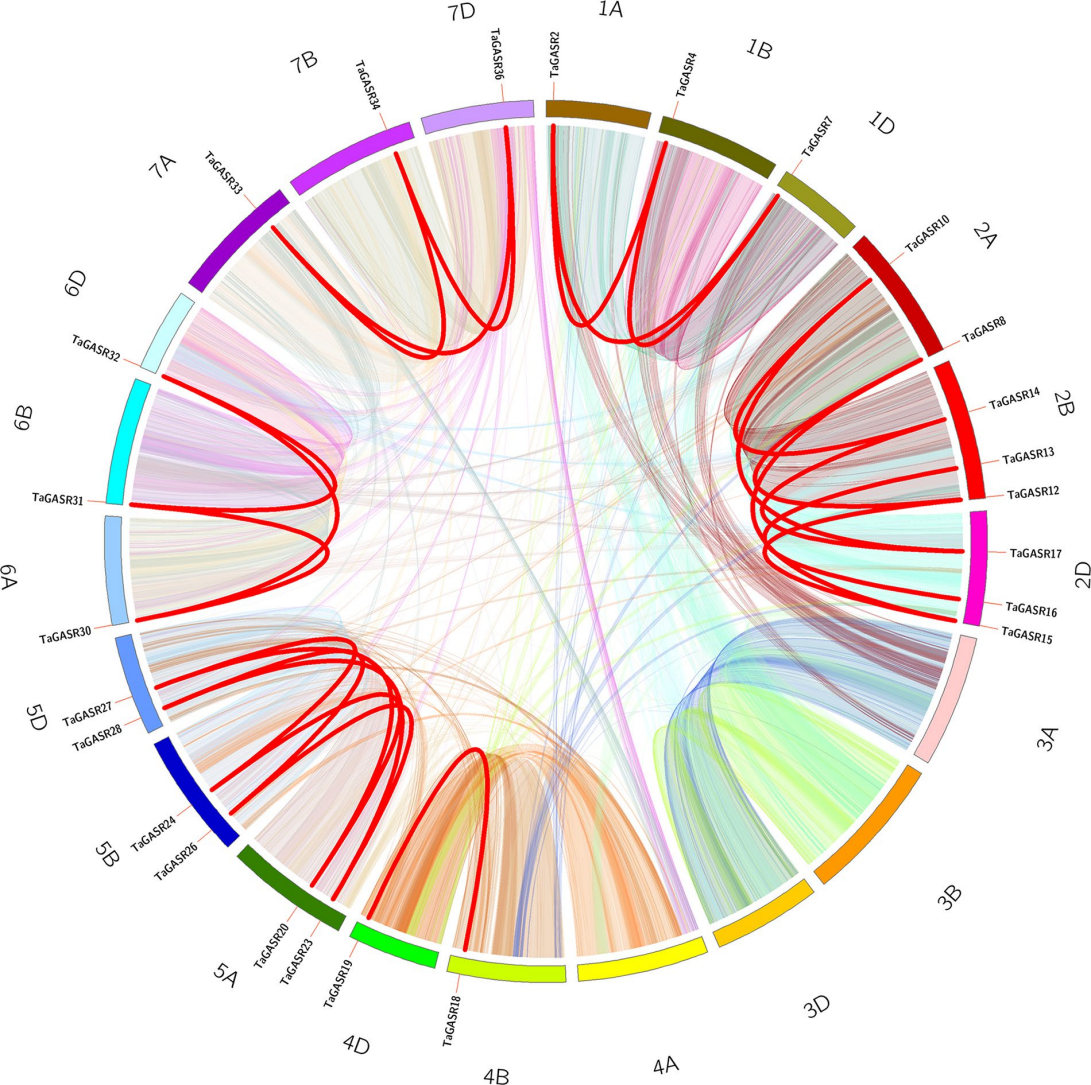
Microsynteny related to TaGASR family in wheat. Wheat chromosomes are shown in different colors. Each chromosome box indicates sequence length in megabases. Different color lines represent syntenic relationships between *TaGASR* regions, whereas thick red lines represent paralogous *TaGASR* genes.

In addition, we identified 25 *TaGASR* genes unevenly distributed on 21 wheat linkage groups (LGs), although no genes were found on LGs 3A, 3B, 3D, and 4A. The most *TaGASR* genes were found in LGs 2B and 2D (3), and some LGs have only one gene (e.g. LG 1A). We also found no significant positive correlation between LG length and the number of *TaGASR* genes (**Figure 6**). Furthermore, we detected 25 pairs of segmentally duplicated genes and seven pairs of tandemly duplicated genes in the 37 genes of the *TaGASR* gene family. These were found to be unevenly distributed on chromosomes 1D, 2A, 2B, 5A, 5B, and 5D (**Table 5**).

### Expression of *TaGASR* Genes During Seed Imbibition

**Figure 7:**
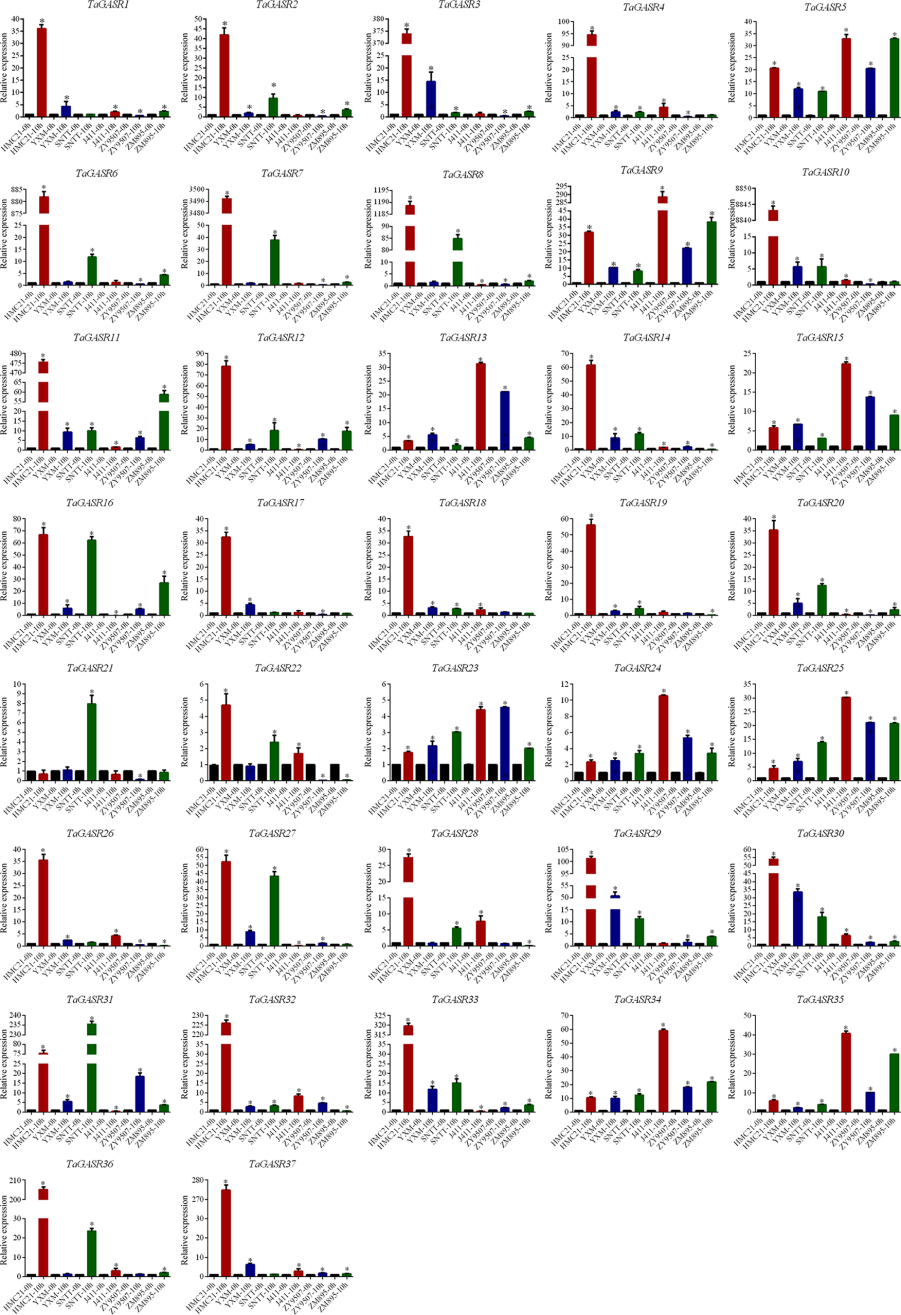
Expression patterns of 37 TaGASR genes during seed imbibition in six wheat varieties. Y-axis: relative expression; Error bars indicate 6 ± SE. *Statistical analysis of materials and methods has been labeled.

The expression patterns of the 37 *TaGASR* genes were investigated at 0 h and 10 h after seed imbibition in six wheat varieties with contrasting seed dormancy phenotypes. After 10 h of imbibition, seeds from three varieties (HMC21, YXM, and SNTT) with high levels of seed dormancy showed no seed germination, whereas seeds from three different varieties (J411, ZY9507, and ZM895) with low levels of seed dormancy showed obvious germination (average GI: 0.97, 0.91, and 0.93, respectively; **Table S9**). Relative to that unimbibed seeds, most of the 37 *TaGASR* genes were up-regulated in response to imbibition, whereas a few were down-regulated or showed no significant differences in gene expression (e.g. *TaGASR21*). For each *TaGASR* gene, we also found obvious differences in relative transcript levels among the six compared wheat varieties. In particular, five specific *TaGASR* genes (*TaGASR15*/-*24*/-*25*/-*34/-35*) were more highly transcribed in the three varieties with low levels of seed dormancy than in the three varieties with high levels of seed dormancy. In contrast, we found the opposite trend among the transcription levels of seven *TaGASR* genes (e.g. *TaGASR10/-14*/-*20*/-*27*/-*29*/-*30/-33*) (**Figure 7**).

### Expression Patterns of *TaGASR* Genes in Response to Exogenous GA, ABA, Low and High Temperature Treatments

**Figure 8:**
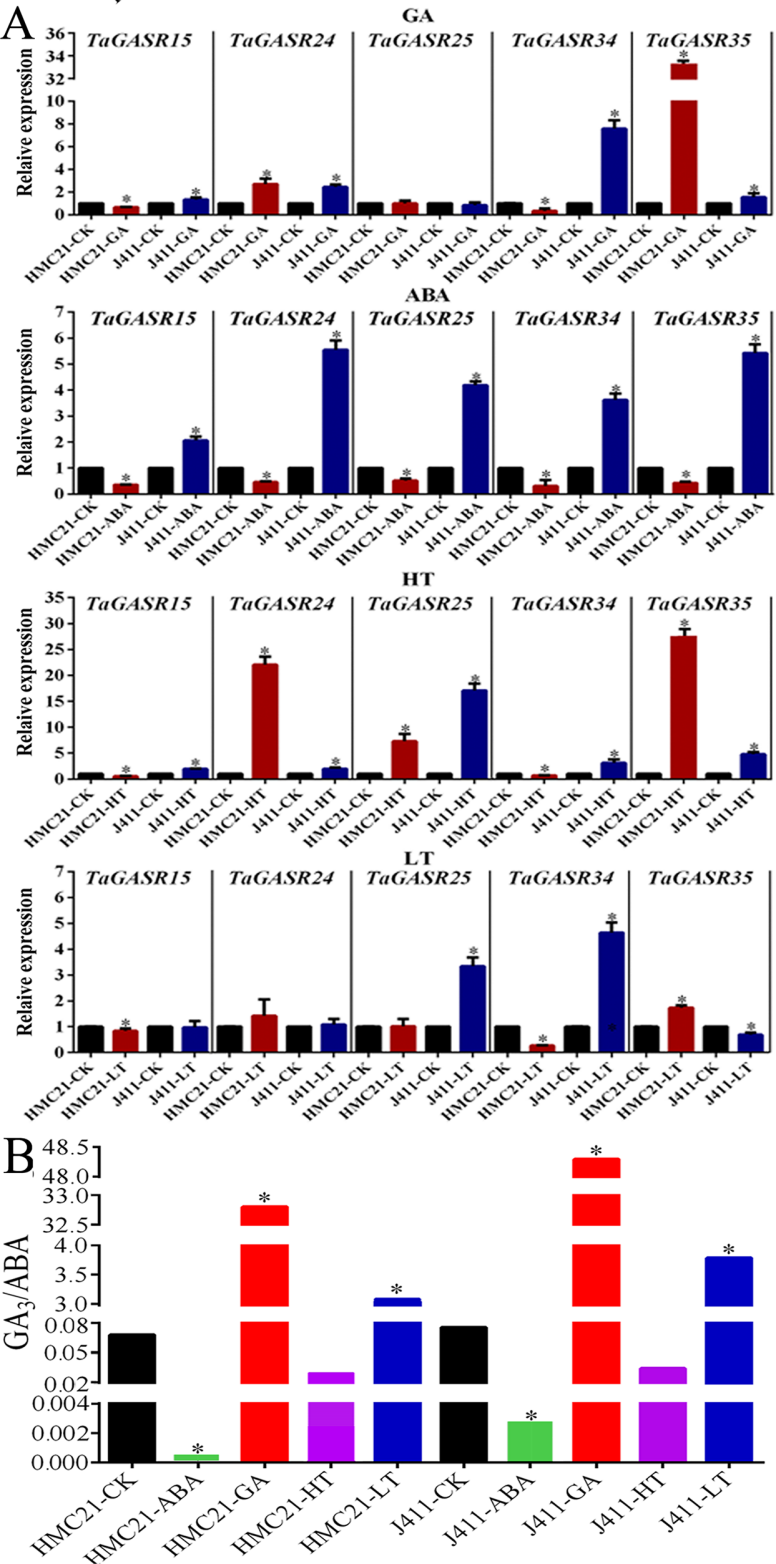
Expression in response to ABA, GA, High Temperature (HT), and Low Temperature (LT) treatments in wheat varieties Jing411 (J411) and Hongmangchun 21 (HMC21). **(A)** The levels of endogenous GA_3_/ABA in J411 and HMC21 **(B)** Expression patterns of five *TaGASR* genes in response to ABA, GA, High Temperature (HT), and Low Temperature (LT) treatments in wheat varieties Jing411 (J411) and Hongmangchun 21 (HMC21). Y-axis: relative expression; Error bars, 6 ± SE.

We further investigated the expression patterns of five *TaGASR* genes (*TaGASR15*/-*24*/-*25*/-*34/-35*) in response to exogenous GA, ABA, low temperature (LT), and high temperature (HT) treatments in varieties HMC21 and J411, which show very high and very low levels of seed dormancy, respectively. Moreover, we assessed the GI values of the two varieties. After 50 µM GA treatment, HMC21 (high dormancy) seeds showed no sensitivity to GA and remained dormant (average GI: 0.00). In contrast, J411 (low dormancy) seeds showed strong sensitivity to GA resulting in high levels of germination (average GI: 0.92; **Table S9**). In addition, in HMC21 and J411 we found different levels of transcription for all five of the *TaGASR* genes examined. Both *TaGASR15* and *TaGASR34* were up-regulated in J411 seeds, but down-regulated in HMC21 seeds. After 50 µM ABA treatment, HMC21 seeds retained strong dormancy (average GI: 0.00), but J411 seeds showed little sensitivity to ABA (average GI: 0.77) (**Table S9**). Moreover, all five genes tested were up-regulated in J411 seeds but were down-regulated in HMC21 seeds. Similarly, after HT (36°C) treatment, HMC21 seeds showed high levels of dormancy (average GI: 0.00), whereas J411 seeds showed low levels of dormancy (average GI: 0.71; **Table S9**). All five genes were also up-regulated in J411 seeds, but *TaGASR15* and *TaGASR34* were down-regulated in HMC21 seeds. After LT (4°C) treatment, HMC21 seeds showed no sensitivity to LT and remained dormant (average GI: 0.00), whereas J411 seeds showed strong sensitivity to LT with high-level germination (average GI: 0.89; **Table S9**). Each of the five genes showed different expression patterns in HMC21 and J411, but only *TaGASR34* was down-regulated in HMC21 seeds yet up-regulated in J411 seeds (**Figure 8A**).

Simultaneously, we also examined the levels of endogenous ABA and GA_3_ in J411 and HMC21 seeds after ABA, GA, HT, and LT treatments, with deionized water as a control. In both J411 and HMC21 seeds, after ABA and HT treatments, the ratios of endogenous GA_3_:ABA were lower compared to control; nevertheless, after GA_3_ and LT treatments, the ratios of endogenous GA_3_:ABA were significantly higher than control. Notably, the ratios of endogenous GA_3_:ABA were consistently lower in HMC21 seeds than in J411 seeds after above four treatments (**Figure 8B**).

Based on the consistent trends between gene expression patterns and corresponding GI phenotypes, we speculated that *TaGASR34* was a candidate gene strongly associated with seed dormancy and germination.

### Cloning and Sequence Analysis of *TaGASR34*

A primer pair (GASR34-7B; **Table S4**) was designed to isolate the *TaGASR34* gene in the J411 and HMC21 varieties. The *TaGASR34* gene was 1,974 bp in length, including a 995 bp promoter sequence, a 458 bp 3′UTR, 3 exons, and 2 introns. Sequence alignment analysis revealed 6 SNP mutations in the *TaGASR34* promoter, and no variation was detected in the *TaGASR34* coding region (**Figure S1**).

In addition, 12 cis-acting elements were identified in the promoter of *TaGASR34*, including one TC-rich repeat element, five MBS (MYB transcription factor binding site) elements, one CE3 element (related to ABA and VP1 response), two Skn-1 elements (related to endosperm expression), two ARE elements, and one box E element. Notably, the replacement of the G/A base at the -16 position resulted in the absence of a box E element (**Figure S2**).

### Validation of the Relationship Between *TaGASR34*, Seed Dormancy, and Seed Germination

All GI phenotypic data showed wide variations within both the NP and CMCC populations across environments, with coefficients of variance of 25.46–55.21% and 38.76–85.79%, respectively (**Table S10**). In NP plants, the average GI value of 13GI15-NP plants was the highest (mean GI = 0.72), ranging from 0.07 to 0.98, followed by the 15GI15-NP (mean GI: 0.64, range: 0.02–0.98) and 13GI5-NP (mean GI: 0.56, range: 0.04–0.91). In CMCC plants, the mean GI values of both the 15GI15-CMCC and 16GI15-CMCC were the highest (mean GI: 0.56), ranging from 0.04 to 0.99 and 0.05 to 0.96, respectively. Significant correlations were detected in GI values assayed under different environments in both NP and CMCC plants, with correlation coefficients of 0.54–0.93 and 0.60–0.95 (*P* < 0.01), respectively (**Table 2**).

**Table 2:** Correlation analysis of seed germination index (GI) phenotypes in NP and CMCC.

Trait	13GI5-NP	13GI15-NP	14GI5-NP	14GI15-NP	14GI5-NP	14GI15-NP
13GI5-NP						
13GI15-NP	0.87**					
14GI5-NP	0.64**	0.61**				
14GI15-NP	0.67**	0.66**	0.93**			
15GI5-NP	0.62**	0.54**	0.66**	0.66**		
15GI15-NP	0.61**	0.57**	0.65**	0.67**	0.85**	
Trait	14GI5-CMCC	14GI15-CMCC	15GI5-CMCC	15GI15-CMCC	16GI5-CMCC	16GI15-CMCC
14GI15-CMCC	0.95**					
15GI5-CMCC	0.83**	0.82**				
15GI15-CMCC	0.76**	0.79**	0.84**			
16GI5-CMCC	0.71**	0.73**	0.77**	0.77**		
16GI15-CMCC	0.60**	0.62**	0.63**	0.74**	0.86**	

**indicates highly significant correlations (p < 0.01).

NP denotes a natural population consisting of 260 wheat varieties; CMCC denotes a mini-core collection of 260 Chinese wheat varieties; 13GI5-NP, 13GI15-NP, 14GI5-NP, 14GI15-NP, 15GI5-NP, and 15GI15-NP represent GI values assayed at 5 and 15 days after harvest in NP; 14GI5-CMCC, 14GI15-CMCC, 15GI5-CMCC, 15GI15-CMCC 16GI5-CMCC, and 16GI15-CMCC represent GI values assayed at 5 and 15 days after harvest in CMCC.

**Figure 9:**
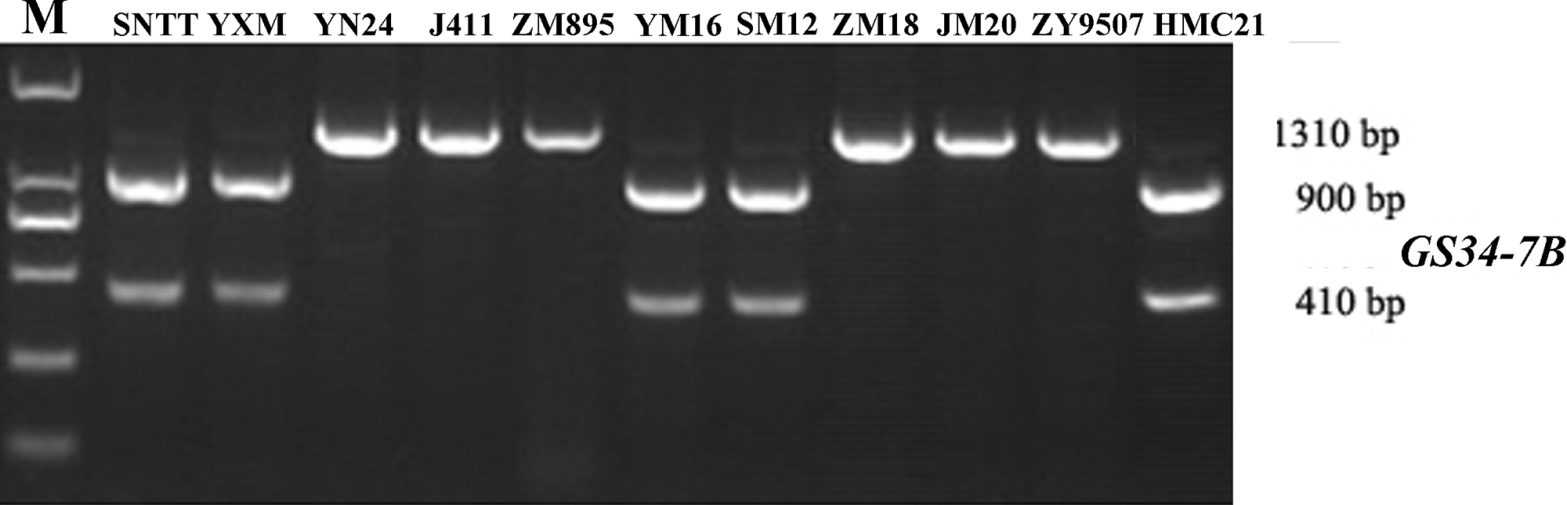
Different genotypes identified by the functional marker GS34-7B in different wheat varieties. Shown are: Suiningtuotuo (SNTT), Yangxiaomai (YXM), Yangnong 24 (YN24), Jing 411 (J411), Zhongmai 895 (ZM895), Yangmai 16 (YM16), Shimai12 (SM12), Zhongmai 18 (ZM18), Jimai 20 (JM20), Zhongyou 9507 (ZY9507), and Hongmangchun 21 (HMC21).

Based on the SNP mutation in the *TaGASR34* promoter listed above, the cleaved amplified polymorphic site (CAPS) marker GS34-7B was developed and used to validate the association between *TaGASR34* and seed dormancy and germination in both CMCC and NP plants. Two allelic variations were identified. These were designated *GS34-7Ba*, which was associated with increasing GI and could be digested into 900-bp and 410-bp fragments, and *GS34-7Bb*, which was associated with decreasing GI and was present as a single undigested 1310-bp fragment (**Figure 9**). In CMCC plants, 224 (86.15% of the total) were found to contain the *GS34-7Ba* allele, whereas 36 (13.85%) carried *GS34-7Bb*. In NP plants, 165 (63.46%) contained the *GS34-7Ba* allele, whereas 95 (36.54%) harbored GS34-7Bb. We detected significant differences (P < 0.01 or 0.05) in mean GI values between varieties with the two alleles of *TaGASR34* in both populations across environments (**Table 3**). Notably, in both CMCC and NP, the frequency distribution of *GS34-7Bb* (13.85% and 36.54%, respectively) was consistently lower than that of GS34-7Ba (86.15% and 63.46%, respectively).

**Table 3:** Descriptive statistics and Mann-Whitney U test results for seed germination index (GI) values between the two alleles of *TaGASR34* in NP and CMCC.

Trait	Genotype	Mean GI (± SD)	Number (%)	Mann-Whitney U Statistic
14GI5-CMCC	*GS34-7Ba*	0.33 ± 0.26	224 (86.15)	3.63**
	*GS34-7Bb*	0.22 ± 0.21	36 (13.85)	
14GI15-CMCC	*GS34-7Ba*	0.51 ± 0.27	224 (86.15)	
	*GS34-7Bb*	0.37 ± 0.25	36 (13.85)	4.13**
15GI5-CMCC	*GS34-7Ba*	0.35 ±0. 25	224 (86.15)	
	*GS34-7Bb*	0.24 ±0. 21	36 (13.85)	3.53**
15GI15-CMCC	*GS34-7Ba*	0.60 ± 0.24	224 (86.15)	
	*GS34-7Bb*	0.50 ± 0.24	36 (13.85)	3.17**
16GI5-CMCC	*GS34-7Ba*	0.52 ± 0.24	224 (86.15)	
	*GS34-7Bb*	0.44 ± 0.22	36 (13.85)	2.88**
16GI15-CMCC	*GS34-7Ba*	0.63 ± 0.19	224 (86.15)	
	*GS34-7Bb*	0.56 ± 0.21	36 (13.85)	2.67**
13GI5-NP	*GS34-7Ba*	0.58 ± 0.18	165 (63.46)	
	*GS34-7Bb*	0.41 ± 0.27	95 (36.54)	3.50**
13GI15-NP	*GS34-7Ba*	0.74± 0.16	165 (63.46)	
	*GS34-7Bb*	0.58 ±0. 25	95 (36.54)	3.25**
14GI5-NP	*GS34-7Ba*	0.37 ± 0.18	165 (63.46)	
	*GS34-7Bb*	0.26 ± 0.24	95 (36.54)	3.13**
14GI15-NP	*GS34-7Ba*	0.47± 0.18	165 (63.46)	
	*GS34-7Bb*	0.36 ± 0.24	95 (36.54)	2.55*
15GI5-NP	*GS34-7Ba*	0.54 ± 0.23	165 (63.46)	
	*GS34-7Bb*	0.42 ± 0.27	95 (36.54)	2.60**
15GI15-NP	*GS34-7Ba*	0.65 ± 0.22	165 (63.46)	
	*GS34-7Bb*	0.53 ± 0.29	95 (36.54)	2.41*

*statistically significant differences in mean GI between alleles (P < 0.05); **highly statistically significant differences in mean GI between alleles (P < 0.01).

NP denotes a natural population consisting of 260 wheat varieties; CMCC denotes a mini-core collection of 260 Chinese wheat varieties; 13GI5-NP, 13GI15-NP, 14GI5-NP, 14GI15-NP, 15GI5-NP, and 15GI15-NP represent GI values assayed at 5 and 15 days after harvest in NP; 14GI5-CMCC, 14GI15-CMCC, 15GI5-CMCC, 15GI15-CMCC 16GI5-CMCC, and 16GI15-CMCC represent GI values assayed at 5 and 15 days after harvest in CMCC.

### Frequency Distribution of *TaGASR34* in Non-Chinese Wheat Germplasms

We also investigated the frequency distribution of *TaGASR34* alleles in 580 wheat germplasms from 23 countries in four continents, including Europe (312), Asia (191), Africa (50), and Oceania (27; **Table S11**). The frequency of *GS34-7Bb* (present in 95 varieties, 16.38% of the total) was significantly lower than the frequency of *GS34-7Ba* (485, 83.62%). On each continent, *GS34-7Bb* also had a consistently lower frequency than *GS34-7Ba* (**Figure S3** and **Table S11**).

## Discussion

### Genome-Wide Identification of *TaGASR* Genes in Common Wheat

Many *GASA* homologs have been studied extensively in a variety of plant species because of their roles in plant development and biotic/abiotic stresses (Taylor and Scheuring, 1994; Ben-Nissan and Weiss, 1996; Aubert et al., 1998; Shi and Olszewski, 1998; Tomoyuki et al., 2006; Zhang and Wang, 2008; Alonso-Ramírez et al., 2009; Zhang et al., 2009; Rubinovich and Weiss, 2010; Zimmermann et al., 2010; Sun et al., 2013; Zhong et al., 2015; Fan et al., 2017). However, little is known regarding *GASR* homologs in common wheat. Only seven *GASR* homologs have yet been identified and characterized. These include *TaGASR7-A1*, which is associated with grain length and weight, and *TaGASR7-B1*, *TaGASR7-D1* (Dong et al., 2014), *TaGAST1*, *TaGAST2*, *TaGAST3*, and *TaGAST4*, all of which are involved in spike/seedling development (Kim et al., 2016).

In this study, we identified 37 *TaGASR* genes and 40/40 paralogous/orthologous pairs in common wheat (**Table S12**). In general, Ka/Ks ratios > 1 indicates accelerated evolution with positive selection, Ka/Ks ratios approximately equal to 1 indicates neutral selection, whereas Ka/Ks ratios < 1 indicates functional constraint by purifying selection (Cui et al., 2019). Here, we found that the Ka/Ks ratio of only one homologous pair was greater than 1, implying that most *TaGASR* genes have undergone negative selection in wheat (**Table 4**). We also identified 27 pairs of *Ta*/*Os* orthologous genes as well as 13 pairs of *Ta*/*At* orthologous genes, suggesting that the genetic relationship between wheat and rice was closer than that between wheat and *Arabidopsis*.

**Table 4:** Estimated divergence times of *TaGASR* genes.

Duplicated *GASR* gene pairs	Ka	Ks	Ka/Ks
*TaGASR1/TaGASR5*	0.00824	0.0698035	0.118045
*TaGASR1/TaGASR6*	0.0284967	0.147842	0.192751
*TaGASR2/TaGASR3*	0.968622	1.09751	0.882562
*TaGASR2/TaGASR4*	0.0261931	0.0269683	0.971256
*TaGASR2/TaGASR7*	0.0137224	0.0287279	0.477669
*TaGASR3/TaGASR7*	0.974121	1.08112	0.901031
*TaGASR4/TaGASR7*	0.0302713	0.0602833	0.502151
*TaGASR5/TaGASR6*	0.0208655	0.0572711	0.364329
*TaGASR8/TaGASR15*	0.0219353	0.272318	0.0805506
*TaGASR9/TaGASR10*	0.0138641	0.0930074	0.149065
*TaGASR9/TaGASR14*	0.0145	0.0727773	0.199238
*TaGASR9/TaGASR17*	0.0102421	0.113233	0.090452
*TaGASR10/TaGASR14*	0.0145488	0.0549615	0.26471
*TaGASR10/TaGASR17*	0.0102831	0.09229	0.111421
*TaGASR13/TaGASR16*	0.0102831	0.09229	0.111421
*TaGASR14/TaGASR17*	0.0112971	0.0368735	0.306375
*TaGASR18/TaGASR19*	0.0140216	0.183682	0.0763361
*TaGASR20/TaGASR24*	0.0171698	0.2001	0.0858063
*TaGASR20/TaGASR27*	0.0322226	0.231381	0.139262
*TaGASR21/TaGASR22*	0.0378267	0.137625	0.274854
*TaGASR21/TaGASR25*	0.0201215	0.188788	0.106583
*TaGASR21/TaGASR28*	0.0196901	0.155897	0.126302
*TaGASR22/TaGASR23*	0.0880006	0.244385	0.36009
*TaGASR22/TaGASR25*	0.0242357	0.169732	0.142788
*TaGASR22/TaGASR26*	0.957339	1.10774	0.864229
*TaGASR22/TaGASR28*	0.0245267	0.108492	0.22607
*TaGASR22/TaGASR29*	0.967446	1.08338	0.892985
*TaGASR23/TaGASR25*	0.0772108	0.294322	0.262334
*TaGASR23/TaGASR26*	0.0195507	0.151985	0.128636
*TaGASR23/TaGASR28*	0.0769464	0.233801	0.329111
*TaGASR23/TaGASR29*	0.0199877	0.109236	0.182978
*TaGASR24/TaGASR27*	0.0279543	0.279131	0.100148
*TaGASR25/TaGASR26*	0.916197	1.22314	0.749054
*TaGASR25/TaGASR28*	0.015762	0.199121	0.0791577
*TaGASR25/TaGASR29*	0.0482323	0.397996	0.121188
*TaGASR26/TaGASR28*	0.944637	1.14526	0.82482
*TaGASR26/TaGASR29*	0.0072859	0.125787	0.0579226
*TaGASR28/TaGASR29*	0.953798	1.12294	0.849379
*TaGASR33/TaGASR36*	0.0211239	0.0004304	49.0828
*TaGASR34/TaGASR36*	0.0083119	0.126995	0.0654507

### Evolutionary and Microanalysis Analysis of *GASR* Genes

Our structural analysis of the 37 *TaGASR* genes revealed varying numbers of exons and introns, indicating that the wheat *GASR* gene family is diverse (**Figure 2B**). Previous studies have reported the number of exons in *GASR* genes from different species ranging from 2 to 5, and the number of introns ranging from 1 to 4. For example, the comparative structures of *GASR* genes in potato and apple suggest stable numbers of introns and exons have been maintained during evolution (Marta et al., 2002; Fan et al., 2017).

During evolution, eukaryotic genomes retain genes and associated regulatory and noncoding sequences on corresponding chromosomes to varying degrees. In the present study, intraspecific microanalysis revealed many collinear genes in wheat (**Figure 6** and **Table 5**), suggesting that the *TaGASR* gene family may have underwent large-scale duplication (e.g. whole-genome or segmental duplication) or tandem duplication events. Structural analysis revealed that segmental duplication was more frequent than tandem duplication in the *TaGASR* gene family. During subsequent evolution, duplicated genes generally experience one of three alternative fates: nonfunctionalization, neofunctionalization, and subfunctionalization (Lynch and Conery, 2000). Many previous studies have reported that gene duplication plays an important role in genome rearrangement and expansion as well as an important role in the generation of gene functional diversity (Zhang et al., 2013; Cui et al., 2019). Together, these results provide a new resource to study the evolution of the *GASR* gene family among different plant species.

**Table 5:** Segmentally and tandemly duplicated *TaGASR* gene pairs.

Gene Name	Gene Name	Duplication Type
*TaGASR2*	*TaGASR4*	Segmental duplication
*TaGASR2*	*TaGASR7*	Segmental duplication
*TaGASR4*	*TaGASR7*	Segmental duplication
*TaGASR12*	*TaGASR8*	Segmental duplication
*TaGASR15*	*TaGASR8*	Segmental duplication
*TaGASR10*	*TaGASR14*	Segmental duplication
*TaGASR10*	*TaGASR17*	Segmental duplication
*TaGASR11*	*TaGASR15*	Segmental duplication
*TaGASR12*	*TaGASR15*	Segmental duplication
*TaGASR13*	*TaGASR16*	Segmental duplication
*TaGASR14*	*TaGASR17*	Segmental duplication
*TaGASR18*	*TaGASR19*	Segmental duplication
*TaGASR20*	*TaGASR24*	Segmental duplication
*TaGASR20*	*TaGASR27*	Segmental duplication
*TaGASR21*	*TaGASR25*	Segmental duplication
*TaGASR21*	*TaGASR28*	Segmental duplication
*TaGASR23*	*TaGASR26*	Segmental duplication
*TaGASR24*	*TaGASR27*	Segmental duplication
*TaGASR25*	*TaGASR28*	Segmental duplication
*TaGASR30*	*TaGASR31*	Segmental duplication
*TaGASR30*	*TaGASR32*	Segmental duplication
*TaGASR31*	*TaGASR32*	Segmental duplication
*TaGASR33*	*TaGASR34*	Segmental duplication
*TaGASR33*	*TaGASR36*	Segmental duplication
*TaGASR34*	*TaGASR36*	Segmental duplication
*TaGASR5*	*TaGASR6*	Tandem duplication
*TaGASR9*	*TaGASR10*	Tandem duplication
*TaGASR11*	*TaGASR12*	Tandem duplication
*TaGASR21*	*TaGASR22*	Tandem duplication
*TaGASR22*	*TaGASR23*	Tandem duplication
*TaGASR25*	*TaGASR26*	Tandem duplication
*TaGASR28*	*TaGASR29*	Tandem duplication

### TaGASR Gene Expression Profiles and Potential Functions

In this study, we found cis-acting regulatory elements responsive to five important plant hormones (ABA, SA, GA, IAA, and MeJA) among the 36 *TaGASR* genes (although not in *TaGASR13*). In addition, we also found three cis-acting regulatory elements that regulate responses to abiotic stress (e.g. drought, low temperature, and defense). In particular, cis-acting regulatory elements associated with drought and low-temperature response were most prevalent among *TaGASR* genes (**Figure 3** and **Table S7**). Taken together, our results suggest that elements responsive to the five plant hormones and elements associated with abiotic stress responses may play important roles in regulating the growth of wheat.

A total of 18 *TaGASR* gene expression profiles were obtained using publicly available microarray data (GSE12508) (Sun et al., 2015). Of these, 72% (13/18) were found to be highly expressed in 22 DAP embryos (22 DAP EM), and 67% (12/18) were highly expressed in anthers before anthesis (Aba). These results indicate that many *TaGASR* genes may play significant roles during wheat growth. We also found that many paralogous gene pairs sharing a high degree of sequence homology had similar patterns of expression (e.g. *TaGASR1*/-*6* and *TaGASR9*/-*10* in 22 DAP EM and Aba plants, as well as *TaGASR14*/-*17*, *TaGASR22*/-*23* and *TaGASR23*/-*29* in PBA and II plants) (**Figure 4** and **Table S8**), implying that paralogous genes may have redundant functions during tissue development (**Figure 4**). These results provide a basis for further investigation of the functions of *TaGASR* genes in wheat.

### Screening of *TaGASR* Genes Associated With Seed Dormancy and Germination and Its Application in Wheat Breeding

The prevalence of PHS in wheat is predominantly due to insufficient dormancy at harvest when seeds are mature (Mares and Mrva, 2001; Ogbonnaya et al., 2008). It is now recognized that moderate to high levels of seed dormancy are required for protection against PHS. Therefore, identification of genes controlling seed dormancy may help to decrease yield losses in wheat caused by PHS. Previous studies have shown that *Arabidopsis AtGASA4*, *AtGASA5* (Rubinovich and Weiss, 2010), and *AtGASA6* (Zhong et al., 2015), as well as *Fagus sylvatica FsGASA4* (Alonso-Ramírez et al., 2009) play key roles in controlling seed dormancy and germination in those two species. However, the roles played by *GASR* homologous genes in wheat are largely unknown.

In this study, we investigated the expression patterns of 37 *TaGASR* genes during seed imbibition in six wheat varieties with contrasting patterns of seed dormancy, and found that the transcript levels of five specific *TaGASR* genes (*TaGASR15*/-*24*/-*25*/-*34*/-*35*) were consistently higher in the three varieties with low dormancy levels than that in the three varieties with high dormancy levels. This suggests that these five *TaGASR* genes may be involved in regulating seed dormancy and germination. In many plant species, seed dormancy and germination are controlled by two major plant hormones (ABA and GA) and temperature (Graeber et al., 2012; Shu et al., 2013; He et al., 2014). Subsequently, we analyzed differences in expression of these genes in varieties J411 and HMC21 following GA_3_, ABA, HT, and LT treatments. We found that only *TaGASR34* was consistently down-regulated in dormant seeds and up-regulated in non-dormant seeds (**Figure 8A**). Also, we analyzed the two endogenous ABA and GA_3_ contents after GA_3_, ABA, HT, and LT treatments, and found that the ratios of endogenous GA_3_:ABA after the above four treatments was consistently lower in HMC21 seeds compared to J411 seeds, which is consistent with the differences in sensitivity of J411 and HMC21 seeds to the above four treatments and their GI phenotypes. These findings indicate that four stress treatments could affect the endogenous hormone levels of the two varieties and thus modulate seed dormancy and germination, which is in accordance with the previous results reported by Yamauchi et al. (2004) (**Figure 8B**). Taken together, this result in combination with GI phenotypic data from different treatments led to speculation that *TaGASR34* may be a candidate gene for the regulation of seed dormancy and germination.

We further isolated the *TaGASR34* gene and found that the G/A replacement of its promoter at the -16 position resulted in the deletion of a box E component. Next, we developed a CAPS marker (GS34-7B) based on the SNP variation. This marker was used to further validate the association of *TaGASR34* with seed dormancy and germination using two natural populations in different environments, suggesting that the allelic version of *TaGASR34* may underlie phenotypic differences in seed dormancy and germination. However, the specific functions of the box E component have not yet been determined, and the detailed regulatory mechanism by which *TaGASR34* is associated with differences in seed dormancy and germination should be explored in future studies.

It is noteworthy that in both Chinese and foreign wheat germplasms, the frequency distribution of the *TaGASR34* allele *GS34-7Bb*, which was associated with higher dormancy levels, was found to be significantly lower than *GS34-7Ba*, which was associated with lower dormancy levels. This result suggests that the favored *GS34-7Bb* allele is not frequently used in wheat breeding.

Previously, Dong et al. (2014) found that a C/G SNP variation at the -3 bp position upstream of the start codon of *TaGASR7-A1* (corresponding to *TaGASR33* identified in this study) affected grain length in common wheat. However, no variation was detected for *TaGASR7-B1* (corresponding to *TaGASR34* identified in this study) or *TaGASR7-D1* (corresponding to *TaGASR36* identified in this study). Zhang et al. (2016) reported that *TaGASR7* was associated with significantly elevated thousand kernel weight (TKW) in *aabbdd* mutant plants with frameshift mutations in all six alleles. Interestingly, our present results indicate that the SNP variation (G/A) at the -16 position of the *TaGASR34* promoter had a significant effect on seed dormancy and germination, however, no effect was observed on thousand grain weight (TGW), grain length (GL) and width (GW) (**Table S13** and **Table S14**). In addition, the presence of different *TaGASR33* alleles had little effect on seed dormancy and germination (data not shown). Therefore, pyramiding the two preferred allelic variants of *TaGASR33* and *TaGASR34* in a single variety may help achieve simultaneous improvement of both grain yield and dormancy.

According to a phylogenetic tree of *GASR* family members, we found that *TaGASR34* was most closely related to the rice homolog *OsGASR7* and the *Arabidopsis* homolog *GASA14*, implying that they may have similar functions. Wang et al. (2009) showed that *OsGSR1* was a positive regulator of GA signaling. Similarly, here we found that *TaGASR34* was up-regulated after GA treatment and showed increased sensitivity to GA, supporting that *TaGASR34* is also involved in GA signaling. However, the role played by *OsGSR1* in regulating seed dormancy and germination is unknown and should be further investigated in future studies. Sun et al. (2013) reported that *Arabidopsis GASA14* expression was up-regulated by GA and down-regulated by transcriptional regulators that repress GA responses, including the DELLA proteins GAI and RGA. The same study also reported that germination rate of the *gasa14-1 GASA14* null mutant was lower than those of Col wild-type plants, thereby further supporting the hypothesis that *TaGASR34* plays a role in regulating seed dormancy and germination.

## Conclusion

In this study, we performed a basic bioinformatics analysis of *TaGASR* gene family in common wheat, and cloned *TaGASR34* as a likely candidate gene involved in the regulation of seed dormancy and germination. Further, we validated the association of *TaGASR34* with seed dormancy and germination, and found the favorable allele *GS34-7Bb* associated with higher seed dormancy was infrequently observed in both Chinese and non-Chinese wheat cultivars and thus had good potential to utilize in wheat PHS resistance breeding. These findings provide a theoretical basis for the subsequent study of *GASR* gene functions in wheat and other crops.

## Data Availability Statement

The genome sequences of wheat, rice and *Arabidopsis* were downloaded from the Ensembl database (http://plants.ensembl.org/index.html), Rice Genome Annotation Project database (http://rice.plantbiology.msu.edu/analyses_search_locus.shtml) and PlantTFDB (http://planttfdb.cbi.pku.edu.cn).

## Author Contributions

XC and SW Conceived the Study, Put Into Effect the Main Bioinformatics Analyses, and Drafted the Manuscript. DX and Xliu Took Part in the Experiments and Drafting of the Manuscript. Xli, WX, JC, HJ, XM, and JW Processed the Experimental Data and Helped to Draft the Manuscript. HZ, CC, JL, and CM Conceived and Guided the Experiments, and Helped in Coordinating the Project and Drafting the Manuscript. All Authors Read and Accepted the Final Manuscript.

## Funding

This work was supported by grants from the National Natural Science Foundation of China (31871608), The National key research and development plan “Breeding new wheat varieties with high-yielding, high-quality and water-saving in the south of Huang-Huai River winter wheat area” — the breeding of new wheat germplasm and varieties with resistance to adversity (2017YFD0100703), The China Agriculture Research System (CARS-03), The National Key Research and Development Program of China (2016YFD0101802, 2017YFD0100804), Wheat Genetics and Breeding Research Platform Innovation Team of Anhui’s University (2015-), Jiangsu Collaborative Innovation Center for Modern Crop Production (JCIC-MCP), and The Agriculture Research System of Anhui province (AHCYTX-02).

## Conflict of Interest

The authors declare that the research was conducted in the absence of any commercial or financial relationships that could be construed as a potential conflict of interest.

## Abbreviations

PHS, Pre-harvest sprouting; GASR, Gibberellin Acid-Stimulated Regulator; SNP, Single nucleotide polymorphism; GI, Germination index; FS, Fielding sprouting; ABA, Abscisic acid; GA, Gibberellin acid; Eth, Ethylene; BR, Brassinolide; IAA, Auxin; ROS, Reactive oxygen species; NO, Nitric oxide; NOS, Nitric oxide synthase; qRT-PCR, quantitative real-time PCR; Ks, number of synonymous substitutions per synonymous site; Ka, number of non-synonymous substitutions per non-synonymous site; CDS, coding sequence; bp, base pair; aa, amino acids; MW, molecular weight; pI, isoelectric point; Da, Dalton.
