# Supplementary material for: Identification and Analysis of the GASR Gene Family in Common Wheat (Triticum aestivum L.) and Characterization of TaGASR34, a Gene Associated With Seed Dormancy and Germination
Source: Front Genet. 2019 Oct 18;10:980. doi: 10.3389/fgene.2019.00980 (PMC6813915; doi:10.3389/fgene.2019.00980)
Supplement: Supplementary file 1 [file DataSheet_1.docx]

Table S1. Names of 260 Chinese varieties of wheat in the Chinese mini-core collection (CMCC).

| Number | Variety | Number | Variety | Number | Variety |
| --- | --- | --- | --- | --- | --- |
| H1 | Neimai11 | H88 | Tanori | H175 | Hongtuozi |
| H2 | Jinmai3 | H89 | Atlas 66 | H176 | Baidatou |
| H3 | Lianglaiyoubaipixiaomai | H90 | Gansu96 | H177 | Jinhuangmai |
| H4 | Bihongsui | H91 | Chaoanxiaomai | H178 | Hongmangmai |
| H5 | Xiaobaimai | H92 | Chike | H179 | Dabaimai |
| H6 | Hongpixiaomai | H93 | Songruimai | H180 | Baiqitou |
| H7 | Dabaipi | H94 | Shengen | H181 | Baimazha |
| H8 | Xiaohongpi | H95 | Shanglinxiaomai | H182 | Laotuotou |
| H9 | Dingxingzhaa | H96 | Kangyou10 | H183 | Gaoyuan506 |
| H10 | Honglidangnianlao | H97 | Taizhong23 | H184 | Ningchun4 |
| H11 | Huoliaomai | H98 | Jinmai2148 | H185 | Jinmai4 |
| H12 | Dahongpi | H99 | Diyouzao | H186 | Dingxi24 |
| H13 | Shanxibaimai | H100 | Jingyang60 | H187 | Huining10 |
| H14 | Mahuaban | H101 | Shite14 | H188 | Shuwang8 |
| H15 | Jiahongmai | H102 | Fuzhuang30 | H189 | Fan6 |
| H16 | Hongjinmai | H103 | Bima1 | H190 | Bimai26 |
| H17 | Baiqimai | H104 | Bima4 | H191 | Guinong10 |
| H18 | Xiaokouhong | H105 | Shijiazhuang54 | H192 | Yunmai34 |
| H19 | Lanhuamai | H106 | Pingyang27 | H193 | Xingyi4 |
| H20 | Daimanghongmai | H107 | Fengchan3 | H194 | Fengmai11 |
| H21 | Zhuoludongmai | H108 | Taishan1 | H195 | Tongjiabaxiaomai |
| H22 | Hongmai | H109 | Jinan2 | H196 | Honghuamai |
| H23 | Laomai | H110 | Youbao | H197 | Baimaizi |
| H24 | Zhongyou9507 | H111 | Bainong3217 | H198 | Chengdouguangtou |
| H25 | Jinmai8 | H112 | Yannong15 | H199 | Jiangmai |
| H26 | Fengkang2 | H113 | Xinong6028 | H200 | Baihuamai |
| H27 | Changzhi6406 | H114 | Shijiazhuang407 | H201 | Hanzhongbai |
| H28 | Beijing8 | H115 | Jimai2 | H202 | Xiaosanyuehuang |
| H29 | Yuandong822 | H116 | Neixiang5 | H203 | Lengtiaohongmai |
| H30 | Luhan328 | H117 | Zhengzhou6 | H204 | Hongxumai |
| H31 | Yanan11 | H118 | Yannong39 | H205 | Zipi |
| H32 | Nongda183 | H119 | Jinan17 | H206 | Baimangmai |
| H33 | Nongda311 | H120 | Xiaoyan6 | H207 | Hongmangzi |
| H34 | Nongda139 | H121 | Shanong7859 | H208 | Yuqiumai |
| H35 | Mingxian169 | H122 | Aifeng3 | H209 | Yangmai |
| H36 | Dongfanghong3 | H123 | Lumai1 | H210 | Yangmai |
| H37 | Xianmai | H124 | Wenmai6 | H211 | Zhushimai |
| H38 | Jiangxizao | H125 | Laizhou953 | H212 | Biantouguangkemai |
| H39 | Honghuazao | H126 | Zhengzhou741 | H213 | Changmangshibiantou |
| H40 | Jiangdongmen | H127 | Baimangmai | H214 | Baidongmai |
| H41 | Dahuangpi | H128 | Huangguaxian | H215 | Chunmai |
| H42 | Chongyanghongmai1 | H129 | Banjiemang | H216 | Hongchunmai (Xinjiangmanasi) |
| H43 | Liuzhutou | H130 | Laolaixia | H217 | Hongjinbaoyin |
| H44 | Shuilizhan | H131 | Luoguding | H218 | Wumangchunmai |
| H45 | Huangshuibai | H132 | Xishanbiansui | H219 | Xindong2 |
| H46 | Baipu (Luoqing) | H133 | Honggoudou | H220 | Kashi1 |
| H47 | Zaoxiaomai | H134 | Baixiaomai | H221 | Kashibaipi |
| H48 | Wangshuibai | H135 | Sanyuehuang | H222 | Tuokexun1 |
| H49 | Yiyuanmai | H136 | Hongqiangchang | H223 | Zhongguochun |
| H50 | Chejianzi | H137 | Youzimai | H224 | Zhengmai9023 |
| H51 | Heshangmai | H138 | Pingyuan50 | H225 | Yanzhan1 |
| H52 | Nuomai | H139 | Baibiansui | H226 | Jiangdongmen |
| H53 | Mangxiaomai | H140 | Baiqimai | H227 | Sumai3 |
| H54 | Sankecun | H141 | Baituozimai | H228 | Afu |
| H55 | Paozimai | H142 | Youmangsagudan | H229 | Ourou |
| H56 | Huadong6 | H143 | Fuyanghong | H230 | Laizhou953 |
| H57 | Liying5 | H144 | Mazhamai | H231 | Fan6 |
| H58 | Sumai3 | H145 | Qiangchangmai | H232 | CS |
| H59 | Yangmai158 | H146 | Huomai | H233 | Zaosui30 |
| H60 | Enmai4 | H147 | Meiqianwu | H234 | Zhenghua0840-3 |
| H61 | Emai6 | H148 | Jianmai | H235 | Lumai19 |
| H62 | Anhui3 | H149 | Sanyuehuang | H236 | Shanhan8675 |
| H63 | Zhemai1 | H150 | Xiaofongshou | H237 | Bolero |
| H64 | Baiyoumai | H151 | Dakoumai | H238 | Pindong904047-9 |
| H65 | Yangmai | H152 | Tumangmai | H239 | Pindong34 |
| H66 | Dunhuachunmai | H153 | Baitiaoyu | H240 | Shangluo81 (2)4-6-6 |
| H67 | Huiqiu | H154 | Dayuhua | H241 | Lankao86 (79)1-2-7 |
| H68 | Daqingmang | H155 | Fumai | H242 | Amazon |
| H69 | Xinkehan9 | H156 | Laoqimai | H243 | ENESCO |
| H70 | Kefeng3 | H157 | Chushanbao | H244 | SW605 |
| H71 | Kelao4 | H158 | Zijuhong | H245 | AC Phil |
| H72 | Xinshuguang1 | H159 | Dalibanmang | H246 | SANGIACOMO |
| H73 | Dongnong101 | H160 | Liuyuehuang | H247 | VAIOLET |
| H74 | Xinshuguang6 | H161 | Gejiaxiang | H248 | SAGITTRIO |
| H75 | Jichun1016 | H162 | Geerhongmai | H249 | Am3 |
| H76 | Chixiaomai | H163 | Motuoxiaomai | H250 | Am9 |
| H77 | Afu | H164 | Bianbachunmai-6 | H251 | GB1 (jing97-995-3) |
| H78 | Gaojiasuo | H165 | Wujiangzhuo | H252 | Hongquanmang |
| H79 | St 2422/464 | H166 | Kangdingxiaomai | H253 | GB3 |
| H80 | Nanda2419 | H167 | Zangdong4 | H254 | GB4 |
| H81 | Orofen | H168 | Rikeze8 | H255 | GB5 |
| H82 | Nonglin10 | H169 | Shanmai (Ningxiayongning) | H256 | GB6 |
| H83 | Shuiyuan86 | H170 | Yizhihua | H257 | GB7 |
| H84 | Early Premium | H171 | Dabaimai | H258 | GB8 |
| H85 | Triumph | H172 | Galaohan | H259 | GB9 |
| H86 | Lovrin 10 | H173 | Shiliyan | H260 | GB10 |
| H87 | Aodesa3 | H174 | Shanmai (Ningxiahelan) |  |  |

Table S2. Names of 260 wheat varieties included in the natural population (NP).

| Number | Variety | Number | Variety | Number | Variety |
| --- | --- | --- | --- | --- | --- |
| G1 | Yangmai158 | G88 | Xingmai6 | G175 | Xing1372 |
| G2 | Annong9267 | G89 | Xingmai13 | G176 | Jingdong10 |
| G3 | Annong92484W | G90 | Yumai7 | G177 | Shixin733 |
| G4 | Wanmai38 | G91 | Yu0926 | G178 | Han5030 |
| G5 | Aizao64 | G92 | Yu09113 | G179 | Henong972 |
| G6 | Zheng9023 | G93 | Neimai8 | G180 | Guan35 |
| G7 | Bainong64 | G94 | Neimai10 | G181 | Heng4338 |
| G8 | Xiaoyan6 | G95 | Neimai11 | G182 | Gaoyou9409 |
| G9 | Glenlen | G96 | Qianmai18 | G183 | Shixin733 |
| G10 | Zhoumai16 | G97 | Qian102032-8 | G184 | Jimai34 |
| G11 | 02P67 | G98 | Qian079984-14 | G185 | Ji5385 |
| G12 | 02Y151 | G99 | Wanke06290 | G186 | Henong326 |
| G13 | Handan6172 | G100 | Xiannong1 | G187 | Han4564 |
| G14 | Aikang58 | G101 | Danshi802 | G188 | Jifeng703 |
| G15 | Zhoumai18 | G102 | Zhongmai1139 | G189 | Heng87-6476 |
| G16 | Y14 | G103 | Junmai35 | G190 | Jinmai31 |
| G17 | Y18 | G104 | Mingtian0417 | G191 | Shigao02-1 |
| G18 | E158 | G105 | Womai0608 | G192 | Cang97-051 |
| G19 | Zhengmai3666 | G106 | Womai8 | G193 | Han9565 |
| G20 | 03G7 | G107 | Yanzhan4110 | G194 | Henong638 |
| G21 | X9610 | G108 | Zhongfan4 | G195 | Weierte1 |
| G22 | Huaimai0360 | G109 | Xinong622 | G196 | Jinhe0459 |
| G23 | Zhou98165 | G110 | Huapei8 | G197 | Jinfeng0459 |
| G24 | Xinmai18 | G111 | Luo9920 | G198 | Shixin618 |
| G25 | Xinong889 | G112 | Fengdecunmai5 | G199 | Hengyou18 |
| G26 | Neixiang203 | G113 | Guanmai1 | G200 | Cangmai119 |
| G27 | Huaimai0320 | G114 | Guomai10 | G201 | Heng7228 |
| G28 | Xinmai19023 | G115 | Jinli88 | G202 | Gaoyou9415 |
| G29 | Jimai19 | G116 | Xinmai0401 | G203 | Xing05-4241 |
| G30 | Jimai20 | G117 | Zhengyumai518 | G204 | Hanyou1 |
| G31 | ENESCO | G118 | Zhongyou989 | G205 | Gaoyou1817 |
| G32 | FARO | G119 | Baofeng10-82 | G206 | Heng6149 |
| G33 | Shijiazhuang8 | G120 | Huaimai0882 | G207 | Gaomai119 |
| G34 | Lankao298 | G121 | Xumai9074 | G208 | Shimai16 |
| G35 | Fanmai5 | G122 | Luyuan502 | G209 | Baiyingdong2 |
| G36 | Annong0807 | G123 | Luo2267 | G210 | Jingxiao-1 |
| G37 | Tai10604 | G124 | Zhoumai31 | G211 | Henongkangbai4 |
| G38 | Niavt14 | G125 | Huarui0712 | G212 | Baomai8 |
| G39 | Guinong775 | G126 | Guoshengmai1 | G213 | Shimai15 |
| G40 | AR2 | G127 | Wanke09636 | G214 | Baomai3 |
| G41 | 03-885 | G128 | Shannong055843 | G215 | Gaocheng8901 |
| G42 | R146 | G129 | Fu0382 | G216 | Zhengmai366 |
| G43 | Jinmai73 | G130 | Bolunxuan182 | G217 | Zhengmai98 |
| G44 | Chuanmai42 | G131 | Huaishi0806 | G218 | Jimai035037 |
| G45 | Mianmai37 | G132 | Xinyuanmai04130 | G219 | Jimai5319 |
| G46 | CP02-63-13-1 | G133 | Lemai091156 | G220 | Zhongmai155 |
| G47 | CP02-8-5-6-1 | G134 | Annong1106 | G221 | Shannong15 |
| G48 | CP02-9-3-1-1-1 | G135 | Annong1107 | G222 | S038186 |
| G49 | CP20-39-11-1 | G136 | Annong1108 | G223 | Zheng004 |
| G50 | Shimai12 | G137 | Annong1110 | G224 | Jimai056487 |
| G51 | Shiyou17 | G138 | Annong1114 | G225 | Jimai7251-1 |
| G52 | Jishi02-1 | G139 | Annong1116 | G226 | Jimai7251-2 |
| G53 | Ji5265 | G140 | Lunxuan988 | G227 | Yumai2 |
| G54 | Bainongaikang58 | G141 | Bainong207 | G228 | Ji95-6023 |
| G55 | Annong1014 | G142 | Annong1039 | G229 | Zhengnong19 |
| G56 | Annong8455 | G143 | Fanmai8 | G230 | Xu5034 |
| G57 | Xuke1 | G144 | Zhongmai895 | G231 | Yannong24 |
| G58 | 984121 | G145 | Huaimai05155 | G232 | Lumai23 |
| G59 | Henong825 | G146 | Luo3429 | G233 | Taishan23 |
| G60 | Zhoumai25 | G147 | Tianming198 | G234 | Linmai2 |
| G61 | Zi0706 | G148 | Annong0942-13 | G235 | Jining16 |
| G62 | Wannong606 | G149 | Hengguan35 | G236 | Wennong6 |
| G63 | Longke0901 | G150 | Su553 | G237 | Zhoumai20 |
| G64 | Aifengzao8 | G151 | Yannong24 | G238 | Zhoumai25 |
| G65 | Su853 | G152 | Yan2415 | G239 | Zhoumai19 |
| G66 | Hongwan3 | G153 | Yannong19 | G240 | Xinong979 |
| G67 | Wanke08585 | G154 | Sumai3 | G241 | Zhoumai17 |
| G68 | Wankenmai081 | G155 | Langzhongbaimaizi | G242 | Zhoumai11 |
| G69 | Dangmai2 | G156 | Peilingxuxumai | G243 | Xin19 |
| G70 | Dinghong208 | G157 | Zitongnuermai | G244 | Luomai21--1 |
| G71 | Bainong3271 | G158 | Suiningtuotuomai | G245 | Luomai21--2 |
| G72 | Yangmai16 | G159 | Wanxianbaimaizi | G246 | Zhoumai23 |
| G73 | Annong0932 | G160 | Wangshuibai | G247 | Huapei0616H-119 |
| G74 | Annong0942 | G161 | Baohuomai | G248 | Jinnong4 |
| G75 | Annong1001 | G162 | Huangguaxian | G249 | Gaoyou9618 |
| G76 | Tai18 | G163 | Chadinghongmai | G250 | Ji95-5219 |
| G77 | Yunong69 | G164 | Tuhuluotou | G251 | Shimai14 |
| G78 | Zhongmai1187 | G165 | Huluotou | G252 | Heng97-4119 |
| G79 | Zhongyu1026 | G166 | Baitutou | G253 | Jinfeng6164 |
| G80 | Zhongyu1095 | G167 | Baiyuhua | G254 | M010 |
| G81 | Luo6099 | G168 | Waitoubai | G255 | M013 |
| G82 | Xinmai23 | G169 | Xiaoyuhua | G256 | M015 |
| G83 | Xinmai0208 | G170 | Baipi224 | G257 | M019 |
| G84 | Yangmai20 | G171 | Baimanghong | G258 | M040 |
| G85 | Yangmai19 | G172 | Yuguo | G259 | M046 |
| G86 | Taikong6 | G173 | Jinmai41 | G260 | M051 |
| G87 | Yangnuomai1 | G174 | Jinmai60 |  |  |

Table S3. qRT-PCR primers for *TaGASR* genes

| TaActin-F | CTTGTATGCCAGCGGTCGAAC |
| --- | --- |
| TaActin-R | CTCATAATCAAGGGCCACG |
| TaGASR1F | AAGCTCACGCCGTCGATTGC |
| TaGASR1R | CCGTCGTGCGTGGTCATGTG |
| TaGASR2F | GGCTTGTGTCGCACGCACTC |
| TaGASR2R | GACGTAGCTGAGCAACGGATCTTG |
| TaGASR3F | ACCGGCTGGTGCAGAAGGATC |
| TaGASR3R | GGCACCTCTTGCAGCAGTAGTTAC |
| TaGASR4F | TTGTGTCGCACGCACTCTTCC |
| TaGASR4R | TGTGACTTAGCTGAGCAACGGATC |
| TaGASR5F | AAGCTCACGCCGTCGATTGC |
| TaGASR5R | CCGTCGTGCGTGGTCATGTG |
| TaGASR6F | TCCTCCTGCTCCTTGTCGAG |
| TaGASR6R | ACGCCCTGTTGCACAGATTC |
| TaGASR7F | GCTATTGTCGCACGCACTCT |
| TaGASR7R | CTGCACCAGCCGGTTTGTAT |
| TaGASR8F | CCAGGCAACCCGTAACGAAT |
| TaGASR8R | AACTCTGGCATGCACTCACG |
| TaGASR9F | CATCAAGGCTGCCGACTCTC |
| TaGASR9R | CGCCCACCCACAGTCTTTAG |
| TaGASR10F | TGGCATCTCTCCTCCTGCTC |
| TaGASR10R | CCACCCGCAGTCTTTAGAGG |
| TaGASR11F | CAATGCTCGTTGCTCGCAGAAC |
| TaGASR11R | GACGCAGTTACACTTGTCGCAAC |
| TaGASR12F | GCGTCCTGCAAGCCTGTCATC |
| TaGASR12R | GTTCCTCCAGTTCTTCGAGCAACG |
| TaGASR13F | TCTTCCTCGTCGCCTCGTACC |
| TaGASR13R | CACTTGCCGCAGCACATCATG |
| TaGASR14F | CCCCTCTAAAGACTGCGGGT |
| TaGASR14R | CAGTCGGTGTAGCACTTGCC |
| TaGASR15F | CTCTTCCTCCTCGTCGCTCTCC |
| TaGASR15R | TTCTTCCAGTTCTTCGCGCATCG |
| TaGASR16F | GATGGATCTTGACGAGCCGAGTG |
| TaGASR16R | TGCGAGAAGACGTTACATGGAAGC |
| TaGASR17F | CCCCTCTAAAGACTGCGGGT |
| TaGASR17R | CAGTCGGTGTAGCACTTGCC |
| TaGASR18F | CCTGCTCCTCCTCTTGCTCGTC |
| TaGASR18R | CGCACTCCTCGCAACACAGC |
| TaGASR19F | ATGAGCAAGCCATCGAGGTG |
| TaGASR19R | CACAGCCCGCAGTACTTCAT |
| TaGASR20F | GCAGCGACACCAAGTACAGGAAG |
| TaGASR20R | GTCGTTGTAGCAGCCGCACTC |
| TaGASR21F | CCTCCTCTTAGCGTCCTCGTCTC |
| TaGASR21R | TCGCCTTGGAGCACCTCACC |
| TaGASR22F | CTGTGGCTCTCCTCCTCTTCCTC |
| TaGASR22R | GCTGTCGCAGAACGCTGATCC |
| TaGASR23F | ACCACTCTCGCTCTCATTCTCCTC |
| TaGASR23R | GCTGTCGCAGAACGCTGATCC |
| TaGASR24F | GCAGCGACACCAAGTACAGGAAG |
| TaGASR24R | GTCGTTGTAGCAGGCGCACTC |
| TaGASR25F | TCCTCTTGGCATCCTCGTCTCTG |
| TaGASR25R | TCGCCTTGGAGCACCTCACC |
| TaGASR26F | AGCCAAGCAACTCAAGAACACCAC |
| TaGASR26R | TAGGAGGACGAGGAGGAGAAGGAG |
| TaGASR27F | GCCGCAGAGCACGAGGATAATG |
| TaGASR27R | GGCAGGTGTCGCAGCACTTG |
| TaGASR28F | TCCTCTTGGCATCCTCGTCTCTG |
| TaGASR28R | GCACTCGGCGCAGCATATCC |
| TaGASR29F | AGAGCCAAGCAACTCAAGAACACC |
| TaGASR29R | TAGGAGGACGAGGAGGAGAAGGAG |
| TaGASR30F | ACATCTGCTGCGGCAAGTGC |
| TaGASR30R | TGGTGTTCTTCATGTTGGCGTAGC |
| TaGASR31F | TGAGAGGTAGACCGAAGCTCCATC |
| TaGASR31R | TCCAACCACACTGCTTCCAACAC |
| TaGASR32F | GCGTCGTCCTCCTTGTTGTCG |
| TaGASR32R | TGCACTTGCCGCAGCAGATG |
| TaGASR33F | TCGGGGACGCAGTACAAGAA |
| TaGASR33R | CCTCCCTCCTTGGTCTTCCA |
| TaGASR34F | GGGACGCAGTACAAGAAGGC |
| TaGASR34R | GTTGTTGTAGCAGGGGCAGG |
| TaGASR35F | TGATGCTCCTCCTCGCTCTCG |
| TaGASR35R | CGTTGCACATCTCCTTCCTCCAG |
| TaGASR36F | TTCCTCTTCGTGGCGCTCCTC |
| TaGASR36R | CAGCACTTGTTGCAGTAGGTCAGG |
| TaGASR37F | TTCTCACTCGTCGCCGTCTCC |
| TaGASR37R | GCAGAAGAGGAAGCAGCAGCAG |

Table S4. Primer pairs for cloning the full-length *TaGASR34* gene and its functional marker

| Marker | Forward / Reverse primer sequence |
| --- | --- |
| GASR34-7B | F: GAGGGCCGGAGATAAATAG |
|  | R: CTGCGGTAAGTGGTCGTT |
| GS34-7B | F: ACACCTCGGTTCAATGCC |
|  | R: CCTTCTTGTACTGCGTCCC |

Table S5. Sequence alignment of *TaGASR34* in the J411 (J411) and Hongmangchun 21 (HMC21) varieties.

HMC21 GAGGGCCGGAGATAAATAGTGTTGGGTGCCTTTATGTATCTTCTCCAAATAAATGTCTGA 60

J411 --------------------tc-------------------g------------------ 60

HMC21 GATGGGAGCGGCCCACACCTCGGTTCAATGCCCCGCCCCTGCCTTGCAGGCCATCTACTG 120

J411 ---------a-------------------------------------------------- 120

HMC21 CGCATGTCAGTTGTAAAAATACGCAGCTATACAGATTGTTATATGGGGGTTGAAATCATT 180

J411 ------------------------------------------------------------ 180

HMC21 ATACGGCTCACGCGTATTGAAGTTGCACGAATCACGCGAGTACATGCGGTGTGGATACCA 240

J411 ------------------------------------------------------------ 240

HMC21 GCCGCGCGTGGACGCATGTCCACTTGCGATTATTCGCGTTATTGCTATATATACGTACAA 300

J411 ------------------------------------------------------------ 300

HMC21 ACTCATGACAATTATGTATCAACAACAATAAATACTCTAGTAACTGAATTTTGCCCAGTA 360

J411 ------------------------------------------------------------ 360

HMC21 AAACTTTGCTACAGATGAAACTAAAACAAGCCCTATATGACTATGCAACCTGTGGTTATC 420

J411 --------------------------------------------------a--------- 420

HMC21 TGCTAACGAGCTTTGTCTTTATCTTTTAATCTAACATAACACACAAAGTGTGGTTTTGTT 480

J411 ------------------------------------------------------------ 480

HMC21 CTAGTACGGCAGTTATTGTTCGCAAAACCAAGCAAAATATGAATGTTGGCGTTGCCAAGA 540

J411 --------a--------------------------------------------------- 540

HMC21 GTCCAACTTCTTTTCCTAGAAGAGATATTGCTTTTGCCGATTGTGATCCCATATATTTTT 600

J411 ------------------------------------------------------------ 600

HMC21 ATCAGAAATCTGCCTACTCACGGTCACGGTTATAGTATCACCTACCCTCGGCACAGCACA 660

J411 ------------------------------------------------------------ 660

HMC21 AAATGCAAAAGCAAGAGCCTTCCCACGGAAAAAGAGAGCAGGGTCGCGGTGCACTGCAGG 720

J411 ------------------------------------------------------------ 720

HMC TCTGTAACGCATATGCATGGTCAGAGCAGCCAGTGGAGCATCACATGCCGAGTACAGACA 780

J411 ------------------------------------------------------------ 780

HMC21 GCAGGAGCAGGCCCGTCCTCTCCACAGTAACGCTTCCATAACTGCTCACCCCCCACCACC 840

J411 ------------------------------------------------------------ 840

HMC21 GTCCACCACCTCTCTCCCCCGCCTCGTGCCCAGCTAGCTGCCCTTCCTCCTCTACATATA 900

J411 ------------------------------------------------------------ 900

HMC21 CCCAGGCGACCCAACCAAAAGATCTCACCTCTCAAGTCCACAATTCACATCGCTCTTACC 960

J411 ------------------------------------------------------------ 960

HMC21 CAGCCACACGCGGAGAAGAGACCCAGCAAGCGCCATGGCCAAGATCTCCTTCCTCCTCGT 1020

J411 ----------------------g------------------------------------- 1020

HMC21 GGCGCTCCTCGTCCTCGCCGTCGCGTTCCCCGTGGTAATCACCCATGCCACGTCGCCTCT 1080

J411 ------------------------------------------------------------ 1080

HMC21 CTCCTTCATCCTTCAGCCATGCATTCATATGTTTTTTTCCTTCACTCACCGGTGCTCTTC 1140

J411 ------------------------------------------------------------ 1140

HMC21 TCCTTTGGGCCGTGCCGTGCAGGAGGTGATGGGAGGCGGCAACGGCGGCGCCGGCGGCGG 1200

J411 ------------------------------------------------------------ 1200

HMC21 CGGCAAGCTCAAGCCATGGGGTAAACCCACTCGCACTCGCAGTATTGCTAGCGCATTTCG 1260

J411 ------------------------------------------------------------ 1260

HMC21 CCGCGTTTCTTCTAGCCTGGTCCTTTTGTTTTTTTGTAAAAAAGAAACTGACGTTTTGGG 1320

J411 ------------------------------------------------------------ 1320

HMC21 GGCGTTTGTGCAGAGTGCTCGTCCAAGTGCTCGTCGCGGTGCTCGGGGACGCAGTACAAG 1380

J411 ------------------------------------------------------------ 1380

HMC21 AAGGCGTGCCTGACCTACTGCAACAAGTGCTGCGCCACTTGCCTCTGCGTGCCGCCGGGC 1440

J411 ------------------------------------------------------------ 1440

HMC21 ACCTACGGCAACAAGGGCGCCTGCCCCTGCTACAACAACTGGAAGACCAAGGAGGGAGGC 1500

J411 ------------------------------------------------------------ 1500

HMC21 CCCAAGTGCCCCTAGATTCTTGATTTTCTTTCTTCTTCTTCTGGGGTGCCAGCTTGCGGT 1560

J411 ------------------------------------------------------------ 1560

HMC21 TGATGGTTATTCACTGCTCGGCCATCAAAATGTACTACAGTAGATCTGAATTATGTGATG 1620

J411 ------------------------------------------------------------ 1620

HMC21 GGCATTTAATCAGTGGCATGTGAATTGCCCTCCCAGTTACCTGTATTTCTATCAGTAAGA 1680

J411 ------------------------------------------------------------ 1680

HMC21 TGTGGAAAACTGGAGGCACTCCGCCACTCCCACATGATTATAGTGGGACCTATCGAGCTT 1740

J411 ------------------------------------------------------------ 1740

HMC21 TATTGTTCCTTGTGCGCCTGTGCACCGTGCTTTCTTCTTGTCTCAAAAGTCAGATGAAGC 1800

J411 ------------------------------------------------------------ 1800

HMC21 ATGAAGCGCCCGTACCATTCGGCATGAATGATGAGCTATCACAATGAACAAAAGCATGCA 1860

J411 ------------------------------------------------------------ 1860

HMC21 ACGCCCGTTCATCTTTCTTTCGTTTTCAGATGTGCAACGAGCATCTTTTGTTGTTATTGG 1920

J411 ------------------------------------------------------------ 1920

HMC21 TACTGAGGCCATCCAAATGGATCATGCAGACACTAAAACGACCACTTACCGCAG 1974

J411 ------------------------------------------------------ 1974

Table S6. Detailed information regarding predicted *AtGASR* and *OsGASR* genes

| **Name** | **Gene ID** | **Location** | **ORF length (bp)** | **Size (aa)** | **MW (Da)** | **pI** |
| --- | --- | --- | --- | --- | --- | --- |
| *AtGASR1* | AT1G10588 | Chr1:3501202-3501904 | 273 | 90 | 9,809.32 | 7.41 |
| *AtGASR2* | AT1G22690 | Chr1:8027294-8028125 | 360 | 119 | 12,938.94 | 9.52 |
| *AtGASR3* | AT1G74670 | Chr1:28053286-28054149 | 306 | 101 | 11,341.38 | 9.01 |
| *AtGASR4* | AT1G75750 | Chr1:28441526-28442367 | 297 | 98 | 10,744.66 | 9.4 |
| *AtGASR5* | AT2G14900 | Chr2:6404175-6405330 | 327 | 108 | 11,381.36 | 8.74 |
| *AtGASR6* | AT2G18420 | Chr2:7993801-7994554 | 285 | 94 | 10,154.81 | 8.67 |
| *AtGASR7* | AT2G30810 | Chr2:13127826-13128666 | 321 | 106 | 11,671.47 | 7.96 |
| *AtGASR8* | AT2G39540 | Chr2:16500866-16501241 | 264 | 87 | 9,440.02 | 8.63 |
| *AtGASR9* | AT3G02885 | Chr3:638021-639055 | 294 | 97 | 10,845.03 | 9.68 |
| *AtGASR10* | AT3G10185 | Chr3:3145579-3146199 | 312 | 103 | 11,372.61 | 8.89 |
| *AtGASR11* | AT4G09600 | Chr4:6072804-6073612 | 300 | 99 | 10,704.61 | 8.88 |
| *AtGASR12* | AT4G09610 | Chr4:6074770-6075645 | 300 | 99 | 10,531.44 | 8.98 |
| *AtGASR13* | AT5G14920 | Chr5:4826479-4827980 | 828 | 275 | 29,138.45 | 9.98 |
| *AtGASR14* | AT5G15230 | Chr5:4944900-4946216 | 321 | 106 | 11,996.20 | 9.46 |
| *AtGASR15* | AT5G59845 | Chr5:24111324-24112020 | 270 | 89 | 9,746.47 | 8.97 |

| **Name** | **Gene ID** | **Location** | **ORF length(bp)** | **Size (aa)** | **MW(Da)** | **pI** |
| --- | --- | --- | --- | --- | --- | --- |
| *OsGASR1* | LOC_Os03g14550 | Chr3:7895245-7895883 | 306 | 101 | 10,968.60 | 8.26 |
| *OsGASR2* | LOC_Os03g41060 | Chr3:22816933-22817775 | 285 | 94 | 9,929.73 | 9.12 |
| *OsGASR3* | LOC_Os03g55290 | Chr3:31464840-31465625 | 282 | 93 | 9,911.59 | 8.77 |
| *OsGASR4* | LOC_Os04g39110 | Chr4:23242283-23243202 | 318 | 105 | 11,127.99 | 8.78 |
| *OsGASR5* | LOC_Os05g31280 | Chr5:18183823-18185191 | 459 | 152 | 15,922.36 | 8.98 |
| *OsGASR6* | LOC_Os05g35690 | Chr5:21198101-21198992 | 279 | 92 | 9,685.45 | 8.86 |
| *OsGSR1* | LOC_Os06g15620 | Chr6:8847702-8848847 | 333 | 110 | 11,308.32 | 9.28 |
| *OsGASR8* | LOC_Os06g51320 | Chr6:31082746-31084111 | 405 | 134 | 14,529.91 | 9.06 |
| *OsGASR9* | LOC_Os07g40240 | Chr7:24125333-24127487 | 309 | 102 | 10,678.64 | 8.78 |
| *OsGASR10* | LOC_Os09g24840 | Chr9:14828981-14830515 | 339 | 112 | 12,149.15 | 9.04 |
| *OsGASR11* | LOC_Os10g02625 | Chr10:1004276-1005490 | 315 | 104 | 11,447.13 | 7.43 |

Table S7. Promoter analysis of the *TaGASR* gene family

| Gene | Biotic stress | | | | | | | | Abiotic stress | | |
| --- | --- | --- | --- | --- | --- | --- | --- | --- | --- | --- | --- |
|  | ABA | MeJA | | Auxin | | SA | GA | | Drought | Low Temp. | Defense |
|  | ABREs | CGTCA-motifs | TGACG-motifs | TGA-elements | AuxRR-cores | TCA-elements | GARE-motifs | P-boxes | MBSs | LTRs | TC-rich repeats |
| *TaGASR1* | 5 |  |  | 1 | 1 |  |  |  |  |  | 1 |
| *TaGASR2* | 2 | 1 | 1 |  |  |  |  | 1 | 1 | 1 |  |
| *TaGASR3* | 4 |  |  |  |  | 1 | 1 |  |  |  | 1 |
| *TaGASR4* |  | 4 | 4 |  |  |  |  | 1 |  |  |  |
| *TaGASR5* | 6 | 3 | 3 |  |  | 1 |  |  |  |  |  |
| *TaGASR6* | 1 |  |  | 1 |  |  | 1 |  | 1 |  |  |
| *TaGASR7* |  | 2 | 2 | 1 |  |  |  | 1 |  |  |  |
| *TaGASR8* | 1 | 5 | 5 | 1 |  |  |  | 1 | 2 |  |  |
| *TaGASR9* | 7 | 4 | 4 | 3 |  | 1 |  |  | 1 |  |  |
| *TaGASR10* | 6 | 1 | 1 |  |  |  | 1 | 1 |  |  |  |
| *TaGASR11* | 1 |  |  |  |  | 1 | 1 |  |  |  | 1 |
| *TaGASR12* | 4 | 1 | 1 |  |  | 1 |  |  |  |  | 1 |
| *TaGASR13* |  |  |  |  |  |  |  |  |  |  |  |
| *TaGASR14* | 1 | 3 | 3 |  |  |  | 1 |  |  |  |  |
| *TaGASR15* |  | 1 | 1 | 1 |  | 1 |  |  | 2 |  |  |
| *TaGASR16* | 4 | 2 | 2 | 1 | 1 |  |  |  |  | 1 | 2 |
| *TaGASR17* | 1 | 4 | 4 | 1 |  | 1 |  |  |  |  |  |
| *TaGASR18* | 4 | 2 | 2 | 1 |  | 1 |  |  | 1 | 1 |  |
| *TaGASR19* | 5 |  |  | 1 |  | 1 |  |  |  |  | 1 |
| *TaGASR20* | 3 | 3 | 3 |  |  | 2 | 1 |  | 2 | 3 |  |
| *TaGASR21* | 1 | 3 | 3 |  |  | 1 |  |  |  |  |  |
| *TaGASR22* | 1 | 3 | 3 | 1 |  | 1 |  |  |  | 1 |  |
| *TaGASR23* | 1 | 3 | 3 | 1 |  | 2 |  |  |  |  |  |
| *TaGASR24* | 3 | 5 | 5 | 1 |  | 1 |  |  | 2 | 4 |  |
| *TaGASR25* | 2 | 2 | 2 |  |  | 1 | 1 |  |  | 1 |  |
| *TaGASR26* | 2 | 2 | 2 |  |  | 1 |  |  |  | 1 |  |
| *TaGASR27* | 1 | 2 | 2 | 1 |  | 1 |  |  |  | 1 |  |
| *TaGASR28* | 3 | 4 | 4 | 3 |  | 1 |  |  |  |  |  |
| *TaGASR29* | 3 | 3 | 3 |  | 1 | 1 |  |  |  | 2 |  |
| *TaGASR30* | 2 | 1 | 1 |  |  | 1 |  |  |  |  |  |
| *TaGASR31* |  | 1 | 1 |  |  | 1 |  |  | 1 |  |  |
| *TaGASR32* |  |  |  | 1 |  |  |  |  |  |  | 1 |
| *TaGASR33* |  |  |  | 1 |  |  |  |  |  |  |  |
| *TaGASR34* |  |  |  | 2 |  | 2 |  |  | 1 |  |  |
| *TaGASR35* | 3 | 1 | 1 |  |  |  |  |  |  | 1 |  |
| *TaGASR36* | 3 |  |  | 1 |  | 2 |  |  | 11 |  |  |
| *TaGASR37* | 4 |  |  |  |  |  |  |  | 1 | 1 |  |

Table S8. Microarray data for *TaGASR* genes

|  | GSC | GSR | GSE | SR | SC | SL | II | FBA | PBA | Aba | 3-5 DAP C | 22 DAP EM | 22 DAP EN |
| --- | --- | --- | --- | --- | --- | --- | --- | --- | --- | --- | --- | --- | --- |
| *TaGASR1* | 5.76584 | 3.27455 | 8.49004 | 4.88559 | 8.40670 | 5.57280 | 9.66099 | 4.40747 | 3.49411 | 11.60184 | 3.79931 | 13.42619 | 7.70194 |
| *TaGASR5* | 3.88297 | 1.15599 | 5.79135 | 3.98533 | 6.02521 | 2.31253 | 6.69283 | 3.45673 | 2.45942 | 12.23540 | 3.41869 | 12.28746 | 5.72145 |
| *TaGASR6* | 5.76584 | 3.27455 | 8.49004 | 4.88559 | 8.40670 | 5.57280 | 9.66099 | 4.40747 | 3.49411 | 11.60184 | 3.79931 | 13.42619 | 7.70194 |
| *TaGASR9* | 6.13445 | 4.67410 | 6.31095 | 4.63399 | 6.81708 | 5.42770 | 6.91468 | 3.83555 | 4.46733 | 5.28101 | 5.99099 | 6.18294 | 4.48319 |
| *TaGASR10* | 6.13445 | 4.67410 | 6.31095 | 4.63399 | 6.81708 | 5.42770 | 6.91468 | 3.83555 | 4.46733 | 5.28101 | 5.99099 | 6.18294 | 4.48319 |
| *TaGASR11* | 7.60021 | 5.93241 | 7.61523 | 6.24178 | 7.60481 | 5.94280 | 9.11280 | 5.69406 | 7.69889 | 10.40932 | 9.51014 | 9.75826 | 8.22718 |
| *TaGASR14* | 6.13445 | 4.67410 | 6.31095 | 4.63399 | 6.81708 | 5.42770 | 6.91468 | 3.83555 | 4.46733 | 5.28101 | 5.99099 | 6.18294 | 4.48319 |
| *TaGASR16* | 7.23007 | 6.53733 | 7.38045 | 5.94732 | 8.14956 | 7.25780 | 9.03378 | 2.94726 | 7.41971 | 10.59873 | 9.75931 | 7.70747 | 5.56758 |
| *TaGASR17* | 6.13445 | 4.67410 | 6.31095 | 4.63399 | 6.81708 | 5.42770 | 6.91468 | 3.83555 | 4.46733 | 5.28101 | 5.99099 | 6.18294 | 4.48319 |
| *TaGASR19* | 9.22013 | 2.77535 | 9.14796 | 3.37387 | 9.20962 | 7.81671 | 10.58487 | 3.01073 | 11.08151 | 3.61587 | 8.46570 | 14.24144 | 8.84364 |
| *TaGASR21* | 9.72954 | 6.60890 | 9.86196 | 6.84287 | 9.07068 | 6.83970 | 10.79693 | 8.35749 | 11.54657 | 10.03105 | 12.54291 | 11.83826 | 9.62286 |
| *TaGASR22* | 10.36788 | 4.93233 | 11.06645 | 3.46411 | 10.13682 | 6.78107 | 12.37829 | 9.85461 | 13.90084 | 10.72863 | 14.11100 | 13.74248 | 10.21715 |
| *TaGASR23* | 10.36788 | 4.93233 | 11.06645 | 3.46411 | 10.13682 | 6.78107 | 12.37829 | 9.85461 | 13.90084 | 10.72863 | 14.11100 | 13.74248 | 10.21715 |
| *TaGASR25* | 10.36788 | 4.93233 | 11.06645 | 3.46411 | 10.13682 | 6.78107 | 12.37829 | 9.85461 | 13.90084 | 10.72863 | 14.11100 | 13.74248 | 10.21715 |
| *TaGASR26* | 10.36788 | 4.93233 | 11.06645 | 3.46411 | 10.13682 | 6.78107 | 12.37829 | 9.85461 | 13.90084 | 10.72863 | 14.11100 | 13.74248 | 10.21715 |
| *TaGASR28* | 10.36788 | 4.93233 | 11.06645 | 3.46411 | 10.13682 | 6.78107 | 12.37829 | 9.85461 | 13.90084 | 10.72863 | 14.11100 | 13.74248 | 10.21715 |
| *TaGASR29* | 10.36788 | 4.93233 | 11.06645 | 3.46411 | 10.13682 | 6.78107 | 12.37829 | 9.85461 | 13.90084 | 10.72863 | 14.11100 | 13.74248 | 10.21715 |
| *TaGASR37* | 11.47029 | 8.11265 | 9.90231 | 7.10437 | 9.81526 | 7.18028 | 10.94678 | 5.26367 | 7.79881 | 6.51028 | 9.92762 | 8.53581 | 7.12701 |

Note: Gene expression on a developmental tissue series for wheat.

GSC: germinating seed, coleoptile; GSR: germinating seed, root; GSE: germinating seed, embryo; SR: seedling, root; SC: seedling, crown; SL: seedling, leaf; II: immature inflorescence; FBA: floral bracts, before anthesis; PBA: pistil, before anthesis; Aba: anthers, before anthesis; 3-5 DAP C: 3-5 DAP caryopsis; 22 DAP EM: 22 DAP embryo; 22 DAP EN: 22 DAP endosperm

Table S9. Seed germination index (GI) values of six wheat varieties in response to different treatments.

| Variety | Stage | GI (Mean±SD) | | | | |
| --- | --- | --- | --- | --- | --- | --- |
|  |  | NT | LT | HT | GA_3_ | ABA |
| J411 | HAI-0h | 0.00 |  |  |  |  |
|  | HAI-10h | 0.97±0.01 |  |  |  |  |
|  | HAI-48h | 0.80±0.02 | 0.89±0.01 | 0.71±0.03 | 0.92±0.02 | 0.77±0.06 |
| ZY9507 | HAI-0h | 0.00 |  |  |  |  |
|  | HAI-10h | 0.91±0.03 |  |  |  |  |
|  | HAI-48h |  |  |  |  |  |
| ZM895 | HAI-0h | 0.00 |  |  |  |  |
|  | HAI-10h | 0.93±0.02 |  |  |  |  |
|  | HAI-48h |  |  |  |  |  |
| HMC21 | HAI-0h | 0.00 |  |  |  |  |
|  | HAI-10h | 0.00±0.01 |  |  |  |  |
|  | HAI-48h | 0.00±0.05 | 0.00±0.02 | 0.00±0.04 | 0.00±0.03 | 0.00±0.06 |
| YXM | HAI-0h | 0.00 |  |  |  |  |
|  | HAI-10h | 0.00±0.03 |  |  |  |  |
|  | HAI-48h |  |  |  |  |  |
| SNTT | HAI-0h | 0.00 |  |  |  |  |
|  | HAI-10h | 0.00±0.02 |  |  |  |  |
|  | HAI-48h |  |  |  |  |  |

Varieties shown include: Zhongmai 895 (ZM895), Jing 411 (J411), Zhongyou 9507 (ZY9507), Yangxiaomai (YXM), Suiningtuotuo (SNTT), Hongmangchun 21 (HMC21). Treatments shown include: NT (normal temperature), LT (low temperature, 4°C), and HT (high temperature, 36°C). HAI-0h, HAI-10h, and HAI-48h represent time points 0, 10, and 48 hours after imbibition, respectively.

Table S10. Descriptive statistics of seed germination index (GI) phenotypes in NP and CMCC plants.

| Trait | Min | Max | Mean | SD | CV (%) |
| --- | --- | --- | --- | --- | --- |
| 13GI5-NP | 0.04 | 0.91 | 0.56 | 0.21 | 37.56 |
| 13GI15-NP | 0.07 | 0.98 | 0.72 | 0.18 | 25.46 |
| 14GI5-NP | 0.00 | 0.80 | 0.35 | 0.19 | 55.21 |
| 14GI15-NP | 0.01 | 0.89 | 0.45 | 0.20 | 43.77 |
| 15GI5-NP | 0.02 | 0.98 | 0.53 | 0.24 | 45.65 |
| 15GI15-NP | 0.02 | 0.98 | 0.64 | 0.24 | 37.71 |
| 14GI5-CMCC | 0.00 | 0.98 | 0.29 | 0.25 | 85.79 |
| 14GI15-CMCC | 0.01 | 0.99 | 0.46 | 0.27 | 59.07 |
| 15GI5-CMCC | 0.01 | 0.95 | 0.31 | 0.24 | 78.24 |
| 15GI15-CMCC | 0.04 | 0.99 | 0.56 | 0.24 | 43.69 |
| 16GI5-CMCC | 0.01 | 0.98 | 0.50 | 0.23 | 47.49 |
| 16GI15-CMCC | 0.05 | 0.96 | 0.56 | 0.21 | 38.76 |

NP represents the natural population consisting of 260 wheat varieties; CMCC represents 260 varieties of Chinese wheat included in the Chinese mini core collection; 13GI5-NP, 13GI15-NP, 14GI5-NP, 14GI15-NP, 15GI5-NP, and 15GI15-NP represent GI values assayed at 5 and 15 days after harvest in NP; 14GI5-CMCC, 14GI15-CMCC, 15GI5-CMCC, 15GI15-CMCC 16GI5-CMCC, and 16GI15-CMCC represent GI values assayed at 5 and 15 days after harvest in CMCC.

Table S11. Genotypes and origins of 580 foreign wheat germplasms

| S. No. | Continent | Country | Name | Genotype | S. No. | Continent | Country | Name | Genotype |
| --- | --- | --- | --- | --- | --- | --- | --- | --- | --- |
| W1 | Asia-1-1 | Afghanistan | unknown | B | W291 | Europe-3-2 | France | Vilmorin 29 | A |
| W2 | Asia-1-2 | Afghanistan | unknown | B | W292 | Europe-3-3 | France | P.M.L.1 | A |
| W3 | Asia-1-3 | Afghanistan | unknown | A | W293 | Europe-3-4 | France | Carré Géant blanc | A |
| W4 | Asia-1-4 | Afghanistan | unknown | A | W294 | Europe-3-5 | France | Bladette de Besplas | A |
| W5 | Asia-1-5 | Afghanistan | unknown | A | W295 | Europe-3-6 | France | Rouge de St-Laud | A |
| W6 | Asia-1-6 | Afghanistan | unknown | B | W296 | Europe-3-7 | France | Roux de Presles | B |
| W7 | Asia-1-7 | Afghanistan | unknown | A | W297 | Europe-3-8 | France | Rouge des Ardennes | A |
| W8 | Asia-1-8 | Afghanistan | unknown | A | W298 | Europe-3-9 | France | Way | A |
| W9 | Asia-1-9 | Afghanistan | unknown | A | W299 | Europe-3-10 | France | Wilson jaune | A |
| W10 | Asia-1-10 | Afghanistan | unknown | A | W300 | Europe-3-11 | France | Rouge de Bordeaux | B |
| W11 | Asia-1-11 | Afghanistan | unknown | A | W301 | Europe-3-12 | France | Parsel | B |
| W12 | Asia-1-12 | Afghanistan | unknown | B | W302 | Europe-3-13 | France | Blé Seigle | B |
| W13 | Asia-1-13 | Afghanistan | unknown | A | W303 | Europe-3-14 | France | Saissette de Maninet P | A |
| W14 | Asia-1-14 | Afghanistan | unknown | B | W304 | Europe-3-15 | France | Maylin | A |
| W15 | Asia-1-15 | Afghanistan | unknown | B | W305 | Europe-4-1 | Greece | unknown | A |
| W16 | Asia-1-16 | Afghanistan | unknown | B | W306 | Europe-4-2 | Greece | Deve | B |
| W17 | Asia-1-17 | Afghanistan | unknown | B | W307 | Europe-4-3 | Greece | unknown | A |
| W18 | Asia-1-18 | Afghanistan | unknown | B | W308 | Europe-4-4 | Greece | Trigonostaro | A |
| W19 | Asia-1-19 | Afghanistan | unknown | B | W309 | Europe-4-5 | Greece | unknown | A |
| W20 | Asia-1-20 | Afghanistan | unknown | A | W310 | Europe-4-6 | Greece | unknown | A |
| W21 | Asia-1-21 | Afghanistan | unknown | A | W311 | Europe-4-7 | Greece | Hokino | A |
| W22 | Asia-1-22 | Afghanistan | unknown | A | W312 | Europe-4-8 | Greece | Vardarka Chervenaka | A |
| W23 | Asia-1-23 | Afghanistan | unknown | A | W313 | Europe-4-9 | Greece | Asprotheri | A |
| W24 | Asia-1-24 | Afghanistan | I.C.A.R.1 | A | W314 | Europe-4-10 | Greece | unknown | A |
| W25 | Asia-1-25 | Afghanistan | unknown | A | W315 | Europe-4-11 | Greece | unknown | A |
| W26 | Asia-1-26 | Afghanistan | unknown | A | W316 | Europe-4-12 | Greece | Zulitsa | A |
| W27 | Asia-1-27 | Afghanistan | unknown | A | W317 | Europe-4-13 | Greece | Arnaout | A |
| W28 | Asia-1-28 | Afghanistan | Sufed Panjabi | A | W318 | Europe-4-14 | Greece | Karabash | A |
| W29 | Asia-1-29 | Afghanistan | unknown | B | W319 | Europe-4-15 | Greece | Vardarka Chervenaka | A |
| W30 | Asia-2-1 | Burma | Shan wheat | A | W320 | Europe-4-16 | Greece | Trigo Obispado | A |
| W31 | Asia-2-2 | Burma | 2193/20-12 | A | W321 | Europe-4-17 | Greece | unknown | A |
| W32 | Asia-2-3 | Burma | 2193/20-14 | A | W322 | Europe-5-1 | Hungary | unknown | A |
| W33 | Asia-2-4 | Burma | 2193/20-8 | B | W323 | Europe-5-2 | Hungary | unknown | A |
| W34 | Asia-3-1 | India | Pusa 80-5 | A | W324 | Europe-5-3 | Hungary | unknown | A |
| W35 | Asia-3-2 | India | Pusa 111 | B | W325 | Europe-5-4 | Hungary | unknown | A |
| W36 | Asia-3-3 | India | Pusa 90 | A | W326 | Europe-5-5 | Hungary | unknown | B |
| W37 | Asia-3-4 | India | unknown | A | W327 | Europe-5-6 | Hungary | unknown | B |
| W38 | Asia-3-5 | India | Gangajali | A | W328 | Europe-5-7 | Hungary | unknown | A |
| W39 | Asia-3-6 | India | Thori | A | W329 | Europe-5-8 | Hungary | unknown | A |
| W40 | Asia-3-7 | India | Boojri | A | W330 | Europe-6-1 | Italy | Mairca di Pali, Flaksberger 19341 | A |
| W41 | Asia-3-8 | India | Dehak | A | W331 | Europe-6-2 | Italy | Flaksberger 21223 | A |
| W42 | Asia-3-9 | India | Dehak | A | W332 | Europe-6-3 | Italy | Gentil Rossa, Flaksberger 19927 | A |
| W43 | Asia-3-10 | India | Dolatkhani | A | W333 | Europe-6-4 | Italy | Flaksberger 21222 | A |
| W44 | Asia-3-11 | India | unknown | A | W334 | Europe-6-5 | Italy | Decimomanna, Flaksberger 20119 | A |
| W45 | Asia-3-12 | India | Gahu (Nepali) or Kyo (Sikkimese) | A | W335 | Europe-6-6 | Italy | unknown | B |
| W46 | Asia-3-13 | India | Desi | A | W336 | Europe-6-7 | Italy | unknown | A |
| W47 | Asia-3-14 | India | Pusa 4 | B | W337 | Europe-6-8 | Italy | Piccolo | A |
| W48 | Asia-3-15 | India | Rodi Garamseli | A | W338 | Europe-6-9 | Italy | Oberdan | A |
| W49 | Asia-3-16 | India | Desi | A | W339 | Europe-6-10 | Italy | unknown | A |
| W50 | Asia-3-17 | India | Pashmak | A | W340 | Europe-6-11 | Italy | unknown | A |
| W51 | Asia-3-18 | India | Pashmak | A | W341 | Europe-7-1 | Poland | Wysololitewka Sobieszynska | A |
| W52 | Asia-3-19 | India | Pusa 6 | A | W342 | Europe-7-2 | Poland | Trigo preto rijo | A |
| W53 | Asia-3-20 | India | unknown | B | W343 | Europe-7-3 | Poland | Bialy Krzyz Ryxa | A |
| W54 | Asia-3-21 | India | Federation | A | W344 | Europe-7-4 | Poland | Hors Concurs | A |
| W55 | Asia-3-22 | India | Thori (beardless) | B | W345 | Europe-7-5 | Poland | Kolben Heinego | A |
| W56 | Asia-3-23 | India | Phundi | A | W346 | Europe-7-6 | Poland | Kitnouska | B |
| W57 | Asia-3-24 | India | unknown | B | W347 | Europe-7-7 | Poland | Kujawianka Wieclawicka | B |
| W58 | Asia-3-25 | India | Ka | A | W348 | Europe-7-8 | Poland | Superelekta Kleszczynskich | A |
| W59 | Asia-3-26 | India | Thori | B | W349 | Europe-7-9 | Poland | Extra Kolben Heinego | A |
| W60 | Asia-3-27 | India | Desi | A | W350 | Europe-7-10 | Poland | Surka Cezostna | A |
| W61 | Asia-3-28 | India | Gahu (Nepali) or Kyo (Sikkimese) | B | W351 | Europe-7-11 | Poland | Ostka Wieclawicka | A |
| W62 | Asia-3-29 | India | Mundia | A | W352 | Europe-7-12 | Poland | Ostka Skomoroska | A |
| W63 | Asia-3-30 | India | Mundia | A | W353 | Europe-7-13 | Poland | Hors Concurs | A |
| W64 | Asia-3-31 | India | Boojri (bearded) | A | W354 | Europe-7-14 | Poland | unknown | A |
| W65 | Asia-3-32 | India | Gahu (Nepali) or Kyo (Sikkimese) | A | W355 | Europe-7-15 | Poland | ferrugineum Al., Flaksberger 17525 | A |
| W66 | Asia-3-33 | India | Boojri | A | W356 | Europe-8-1 | Portugal | Trigo Temporao de Coruche | A |
| W67 | Asia-3-34 | India | Desi White | A | W357 | Europe-8-2 | Portugal | Trigo Precoce | A |
| W68 | Asia-3-35 | India | Boojri (bearded) | B | W358 | Europe-8-3 | Portugal | Trigo Ideal | A |
| W69 | Asia-3-36 | India | Dolatkhani (white) | B | W359 | Europe-8-4 | Portugal | Trigo Candeal | A |
| W70 | Asia-3-37 | India | Lyallpur 14 | A | W360 | Europe-8-5 | Portugal | Trigo mole da regiad | A |
| W71 | Asia-3-38 | India | Lalia | A | W361 | Europe-8-6 | Portugal | Trigo luanco rijo | A |
| W72 | Asia-3-39 | India | White Murga | B | W362 | Europe-8-7 | Portugal | Trigo Colonial | A |
| W73 | Asia-3-40 | India | Soor Ghanum | A | W363 | Europe-8-8 | Portugal | Trigo môcho ruivo | A |
| W74 | Asia-3-41 | India | Dehak | B | W364 | Europe-8-9 | Portugal | Trigo Brancal | A |
| W75 | Asia-3-42 | India | Desi | A | W365 | Europe-8-10 | Portugal | Trigo luanco rijo | A |
| W76 | Asia-3-43 | India | Lyallpur 14 | A | W366 | Europe-8-11 | Portugal | Trigo mole da regiad | A |
| W77 | Asia-3-44 | India | unknown | A | W367 | Europe-8-12 | Portugal | Trigo Marques on Pero Espuna | A |
| W78 | Asia-3-45 | India | Desi | A | W368 | Europe-8-13 | Portugal | Camara's no. 3 | A |
| W79 | Asia-3-46 | India | unknown | A | W369 | Europe-8-14 | Portugal | Trigo Tremês | A |
| W80 | Asia-3-47 | India | unknown | A | W370 | Europe-8-15 | Portugal | Trigo Precoce | A |
| W81 | Asia-3-48 | India | Setwa | B | W371 | Europe-8-16 | Portugal | Trigo Lobeiro | A |
| W82 | Asia-3-49 | India | Sur | A | W372 | Europe-8-17 | Portugal | Trigo Rietti | A |
| W83 | Asia-3-50 | India | Desi White | A | W373 | Europe-8-18 | Portugal | Trigo Rietti | A |
| W84 | Asia-3-51 | India | unknown | B | W374 | Europe-8-19 | Portugal | Trigo Galego | A |
| W85 | Asia-3-52 | India | unknown | B | W375 | Europe-8-20 | Portugal | Trigo Mourisco ruivo | A |
| W86 | Asia-3-53 | India | unknown | A | W376 | Europe-8-21 | Portugal | Trigo Precoce | A |
| W87 | Asia-3-54 | India | unknown | B | W377 | Europe-8-22 | Portugal | Trigo Ribeiro | A |
| W88 | Asia-3-55 | India | unknown | A | W378 | Europe-8-23 | Portugal | Trigo Marquês | A |
| W89 | Asia-3-56 | India | Desi | A | W379 | Europe-8-24 | Portugal | Trigo ruivo fino | B |
| W90 | Asia-3-57 | India | Desi | A | W380 | Europe-8-25 | Portugal | unknown | B |
| W91 | Asia-3-58 | India | Desi | A | W381 | Europe-8-26 | Portugal | Alexandre? (Italian) | A |
| W92 | Asia-3-59 | India | Desi | A | W382 | Europe-8-27 | Portugal | Trigo luanca Bonito | A |
| W93 | Asia-3-60 | India | Desi | B | W383 | Europe-8-28 | Portugal | Trigo Anafil | A |
| W94 | Asia-3-61 | India | Desi | B | W384 | Europe-8-29 | Portugal | Trigo Morenico Ruivo | A |
| W95 | Asia-3-62 | India | Desi | A | W385 | Europe-8-30 | Portugal | Trigo Ideal | A |
| W96 | Asia-3-63 | India | Soor Ghanum | B | W386 | Europe-8-31 | Portugal | Trigo Durasio branco | B |
| W97 | Asia-3-64 | India | Soor Ghanum | B | W387 | Europe-8-32 | Portugal | Trigo Barbela | A |
| W98 | Asia-3-65 | India | Soor Ghanum | A | W388 | Europe-8-33 | Portugal | Trigo Galego Môcho | A |
| W99 | Asia-3-66 | India | Soor Ghanum | A | W389 | Europe-8-34 | Portugal | Trigo Belini | A |
| W100 | Asia-3-67 | India | Soor Ghanum | A | W390 | Europe-8-35 | Portugal | Trigo Ideal | B |
| W101 | Asia-3-68 | India | Lyallpur 10 | A | W391 | Europe-8-36 | Portugal | Trigo Ideal | A |
| W102 | Asia-3-69 | India | Desi White | B | W392 | Europe-9-1 | Romania | A.26 | A |
| W103 | Asia-3-70 | India | Spin | B | W393 | Europe-9-2 | Romania | Tiganesti 653 | A |
| W104 | Asia-3-71 | India | Lalia Desi | A | W394 | Europe-9-3 | Romania | Todiresti | B |
| W105 | Asia-3-72 | India | Desi | A | W395 | Europe-9-4 | Romania | Miercurea Ciucului | B |
| W106 | Asia-3-73 | India | unknown | A | W396 | Europe-9-5 | Romania | Samanta 117 | A |
| W107 | Asia-3-74 | India | Dandi | A | W397 | Europe-9-6 | Romania | unknown | A |
| W108 | Asia-3-75 | India | Sambhoria (Lalwali Wala) | A | W398 | Europe-9-7 | Romania | Samanta 1252 | A |
| W109 | Asia-3-76 | India | Desi | B | W399 | Europe-10-1 | Spain | Mocho | A |
| W110 | Asia-3-77 | India | Dehak Panjabi | B | W400 | Europe-10-2 | Spain | Mocho | A |
| W111 | Asia-3-78 | India | unknown | A | W401 | Europe-10-3 | Spain | Pelado de espiga larga | B |
| W112 | Asia-3-79 | India | unknown | A | W402 | Europe-10-4 | Spain | Gironde de Larrion | B |
| W113 | Asia-3-80 | India | Samna | A | W403 | Europe-10-5 | Spain | Pelado de Estella | B |
| W114 | Asia-3-81 | India | Pusa 12 | A | W404 | Europe-10-6 | Spain | Buen labrador | B |
| W115 | Asia-3-82 | India | Punjab 8A | A | W405 | Europe-10-7 | Spain | Pelado grano cilindrico | A |
| W116 | Asia-3-83 | India | Kaghzi Desi | A | W406 | Europe-10-8 | Spain | Aurora | B |
| W117 | Asia-3-84 | India | unknown | B | W407 | Europe-10-9 | Spain | unknown | A |
| W118 | Asia-3-85 | India | Rustam Exp Farm 79 | A | W408 | Europe-10-10 | Spain | unknown | A |
| W119 | Asia-3-86 | India | Lyallpur 8A | A | W409 | Europe-10-11 | Spain | Mocho Rojo | A |
| W120 | Asia-3-87 | India | Gahu (Nepali) or Kyo (Sikkimese) | A | W410 | Europe-10-12 | Spain | Great wheat | A |
| W121 | Asia-3-88 | India | Asian | A | W411 | Europe-10-13 | Spain | Manitoba | A |
| W122 | Asia-3-89 | India | Gahu (Nepali) or Kyo (Sikkimese) | B | W412 | Europe-10-14 | Spain | unknown | A |
| W123 | Asia-3-90 | India | Walaiti (Mastung wheat) | B | W413 | Europe-10-15 | Spain | Erria | A |
| W124 | Asia-3-91 | India | Desi | A | W414 | Europe-10-16 | Spain | Toseta de Oronz | A |
| W125 | Asia-3-92 | India | C. | A | W415 | Europe-10-17 | Spain | Palado sensible a sequia | A |
| W126 | Asia-3-93 | India | Gahu (Nepali) or Kyo (Sikkimese) | B | W416 | Europe-10-18 | Spain | Great wheat | A |
| W127 | Asia-3-94 | India | Thori | A | W417 | Europe-10-19 | Spain | Rigoctia | A |
| W128 | Asia-3-95 | India | Pusa No 4 | B | W418 | Europe-10-20 | Spain | Moro | A |
| W129 | Asia-3-96 | India | Sufed Panjabi | A | W419 | Europe-10-21 | Spain | Marquis | A |
| W130 | Asia-3-97 | India | Desi | A | W420 | Europe-10-22 | Spain | Trigo blancos | A |
| W131 | Asia-3-98 | India | unknown | B | W421 | Europe-10-23 | Spain | Trigo Candeal | B |
| W132 | Asia-3-99 | India | Desi | A | W422 | Europe-10-24 | Spain | Mocho de Burgos | A |
| W133 | Asia-3-100 | India | Gahu (Nepali) or Kyo (Sikkimese) | A | W423 | Europe-10-25 | Spain | Rico de Ituren | A |
| W134 | Asia-3-101 | India | unknown | A | W424 | Europe-10-26 | Spain | Moro | A |
| W135 | Asia-3-102 | India | unknown | A | W425 | Europe-10-27 | Spain | Mocho Rojo | B |
| W136 | Asia-3-103 | India | Lalmi Faizabad No2 | A | W426 | Europe-10-28 | Spain | Bastanes royo productivo | A |
| W137 | Asia-3-104 | India | Abi | A | W427 | Europe-10-29 | Spain | Galberie | A |
| W138 | Asia-3-105 | India | Abi | A | W428 | Europe-10-30 | Spain | Manitoba velloso | A |
| W139 | Asia-3-106 | India | Pusa 80-5 | A | W429 | Europe-10-31 | Spain | unknown | A |
| W140 | Asia-3-107 | India | Malo | A | W430 | Europe-10-32 | Spain | unknown | A |
| W141 | Asia-3-108 | India | Mundia | A | W431 | Europe-10-33 | Spain | unknown | A |
| W142 | Asia-4-1 | Iran | Autumn wheat | A | W432 | Europe-10-34 | Spain | unknown | A |
| W143 | Asia-4-2 | Iran | unknown | A | W433 | Europe-10-35 | Spain | unknown | B |
| W144 | Asia-4-3 | Iran | unknown | A | W434 | Europe-10-36 | Spain | Barbilla | A |
| W145 | Asia-4-4 | Iran | Razan Ferahan | A | W435 | Europe-10-37 | Spain | Baztanes compacto | A |
| W146 | Asia-4-5 | Iran | unknown | A | W436 | Europe-10-38 | Spain | Rieti navarro, grano lango | B |
| W147 | Asia-4-6 | Iran | unknown | A | W437 | Europe-10-39 | Spain | Catalan de tallo corto | A |
| W148 | Asia-4-7 | Iran | unknown | A | W438 | Europe-10-40 | Spain | Rieti navarro frondoso | A |
| W149 | Asia-4-8 | Iran | unknown | A | W439 | Europe-10-41 | Spain | Catalan royo compacto | A |
| W150 | Asia-4-9 | Iran | unknown | A | W440 | Europe-10-42 | Spain | Royo de dificil desgrane | A |
| W151 | Asia-4-10 | Iran | Kerbelai Ali Khani | A | W441 | Europe-10-43 | Spain | unknown | A |
| W152 | Asia-4-11 | Iran | Sarakhs | A | W442 | Europe-10-44 | Spain | Carpino alto, precoz | A |
| W153 | Asia-4-12 | Iran | unknown | A | W443 | Europe-10-45 | Spain | Carpino Royo de Eslava | A |
| W154 | Asia-4-13 | Iran | Vilayati | A | W444 | Europe-10-46 | Spain | unknown | A |
| W155 | Asia-4-14 | Iran | Sarakhs | A | W445 | Europe-10-47 | Spain | Trigo Obispado | A |
| W156 | Asia-4-15 | Iran | Sarakh | A | W446 | Europe-10-48 | Spain | Hembrilla de la tierra | B |
| W157 | Asia-4-16 | Iran | Serabend | A | W447 | Europe-10-49 | Spain | Catalan de espiga blanca | B |
| W158 | Asia-4-17 | Iran | Safid | A | W448 | Europe-10-50 | Spain | Manitoba | A |
| W159 | Asia-4-18 | Iran | unknown | B | W449 | Europe-10-51 | Spain | Candeal | A |
| W160 | Asia-4-19 | Iran | unknown | A | W450 | Europe-10-52 | Spain | Hembrilla de Isamba | A |
| W161 | Asia-4-20 | Iran | Zardak-i-Harrami | A | W451 | Europe-10-53 | Spain | Rampla | A |
| W162 | Asia-4-21 | Iran | Sarakhsi | A | W452 | Europe-10-54 | Spain | unknown | A |
| W163 | Asia-4-22 | Iran | unknown | A | W453 | Europe-10-55 | Spain | unknown | A |
| W164 | Asia-4-23 | Iran | unknown | A | W454 | Europe-10-56 | Spain | Black Argaña | A |
| W165 | Asia-4-24 | Iran | unknown | A | W455 | Europe-10-57 | Spain | unknown | A |
| W166 | Asia-4-25 | Iran | Ab Mahi | A | W456 | Europe-10-58 | Spain | unknown | A |
| W167 | Asia-4-26 | Iran | Khal Kirmiz | A | W457 | Europe-10-59 | Spain | Marquis | A |
| W168 | Asia-4-27 | Iran | Ab Mahi | A | W458 | Europe-10-60 | Spain | Aduna | A |
| W169 | Asia-4-28 | Iran | Qizilqin Boghda | B | W459 | Europe-10-61 | Spain | Trigo blancos | A |
| W170 | Asia-4-29 | Iran | Khusheh Safid | B | W460 | Europe-10-62 | Spain | Aurora | B |
| W171 | Asia-4-30 | Iran | unknown | A | W461 | Europe-10-63 | Spain | Carpino alto frondoso | A |
| W172 | Asia-4-31 | Iran | Trigo Blocal | A | W462 | Europe-10-64 | Spain | Carpino Royo de Eslava | B |
| W173 | Asia-4-32 | Iran | unknown | A | W463 | Europe-10-65 | Spain | Rieti navarro, tallo fuerte, corto | A |
| W174 | Asia-4-33 | Iran | Catalan de grano corto pesado | A | W464 | Europe-10-66 | Spain | Marzal | A |
| W175 | Asia-4-34 | Iran | Desi | B | W465 | Europe-10-67 | Spain | Pelado de Estello, grano corto | A |
| W176 | Asia-4-35 | Iran | Sukh Das | A | W466 | Europe-10-68 | Spain | Catalan de grano corto | A |
| W177 | Asia-4-36 | Iran | Gahu (Nepali) or Kyo (Sikkimese) | B | W467 | Europe-10-69 | Spain | Hembrilla alto, fuerta | A |
| W178 | Asia-5-1 | Iraq | Rustam Exp Farm 99 | A | W468 | Europe-10-70 | Spain | Royo de alta | A |
| W179 | Asia-5-2 | Iraq | Kandhari, Rustam Exp Farm 859 | A | W469 | Europe-10-71 | Spain | Baztanes de tallos llanos | A |
| W180 | Asia-5-3 | Iraq | Humairah, Rustam Exp Farm 595 | A | W470 | Europe-10-72 | Spain | Catalan de grana corto | B |
| W181 | Asia-5-4 | Iraq | Kandhari, Rustam Exp Farm 860 | A | W471 | Europe-10-73 | Spain | Rieti navarro, alto fuerto | A |
| W182 | Asia-5-5 | Iraq | Lyallpur 11 | A | W472 | Europe-10-74 | Spain | Hembrilla Rieti | A |
| W183 | Asia-6-1 | Turkey | unknown | A | W473 | Europe-10-75 | Spain | Trigo duros | A |
| W184 | Asia-6-2 | Turkey | unknown | A | W474 | Europe-10-76 | Spain | Trigo semi-duro | B |
| W185 | Asia-6-3 | Turkey | unknown | A | W475 | Europe-10-77 | Spain | Jeja reja | A |
| W186 | Asia-6-4 | Turkey | unknown | A | W476 | Europe-10-78 | Spain | Trigo Pinet ó Rochal | A |
| W187 | Asia-6-5 | Turkey | unknown | A | W477 | Europe-10-79 | Spain | Candeal | A |
| W188 | Asia-6-6 | Turkey | unknown | A | W478 | Europe-10-80 | Spain | Rico de Ituren | A |
| W189 | Asia-6-7 | Turkey | unknown | A | W479 | Europe-10-81 | Spain | unknown | A |
| W190 | Asia-6-8 | Turkey | unknown | A | W480 | Europe-10-82 | Spain | Souri | A |
| W191 | Asia-6-9 | Turkey | unknown | B | W481 | Europe-10-83 | Spain | Blé cheliostazo | A |
| W192 | Oceania-1-1 | Australia | unknown | A | W482 | Europe-10-84 | Spain | Vilayati | A |
| W193 | Oceania-1-2 | Australia | unknown | A | W483 | Europe-10-85 | Spain | Richelle Hative | A |
| W194 | Oceania-1-3 | Australia | unknown | A | W484 | Europe-10-86 | Spain | Baztanes feurtes | A |
| W195 | Oceania-1-4 | Australia | Improved Steinwedel | A | W485 | Europe-10-87 | Spain | Trigo barbilla | A |
| W196 | Oceania-1-5 | Australia | Aussie | A | W486 | Europe-11-1 | USSR | erythrospermum Korn., Flaksberger 17517 | A |
| W197 | Oceania-1-6 | Australia | Sultan | A | W487 | Europe-11-2 | USSR | erythrospermum Korn., Flaksberger 23613 | A |
| W198 | Oceania-1-7 | Australia | Sunset | A | W488 | Europe-11-3 | USSR | erythrospermum Korn., Flaksberger 17525 | A |
| W199 | Oceania-1-8 | Australia | Ford | A | W489 | Europe-11-4 | USSR | Desi | A |
| W200 | Oceania-1-9 | Australia | Fife | A | W490 | Europe-11-5 | USSR | Zlotka Miczynskiego | A |
| W201 | Oceania-1-10 | Australia | Early Bird | A | W491 | Europe-11-6 | USSR | Zaborzanka | A |
| W202 | Oceania-1-11 | Australia | Majestic | A | W492 | Europe-11-7 | USSR | Muganka x Belokoloska | A |
| W203 | Oceania-1-12 | Australia | Rajah | A | W493 | Europe-11-8 | USSR | Edda | B |
| W204 | Oceania-1-13 | Australia | Cedar | B | W494 | Europe-11-9 | USSR | Pusa 6 | A |
| W205 | Oceania-1-14 | Australia | unknown | A | W495 | Europe-11-10 | USSR | unknown | A |
| W206 | Oceania-1-15 | Australia | unknown | A | W496 | Europe-11-11 | USSR | Lalmi | A |
| W207 | Oceania-1-16 | Australia | unknown | A | W497 | Europe-11-12 | USSR | unknown | A |
| W208 | Oceania-1-17 | Australia | Union | A | W498 | Europe-11-13 | USSR | Muganka | A |
| W209 | Oceania-1-18 | Australia | Waratah | A | W499 | Europe-11-14 | USSR | unknown | A |
| W210 | Oceania-1-19 | Australia | Canberra | A | W500 | Europe-11-15 | USSR | Valki Exp Sta 3 | A |
| W211 | Oceania-1-20 | Australia | Exquisite | A | W501 | Europe-11-16 | USSR | Armavir | A |
| W212 | Oceania-1-21 | Australia | Federation | A | W502 | Europe-11-17 | USSR | unknown | A |
| W213 | Oceania-1-22 | Australia | Duchess | A | W503 | Europe-11-18 | USSR | Valki Exp Sta 1 | A |
| W214 | Oceania-1-23 | Australia | Teakles Red | A | W504 | Europe-11-19 | USSR | Valki Exp Sta 3 | A |
| W215 | Oceania-1-24 | Australia | King's White | A | W505 | Europe-11-20 | USSR | unknown | A |
| W216 | Oceania-1-25 | Australia | unknown | B | W506 | Europe-11-21 | USSR | unknown | A |
| W217 | Oceania-1-26 | Australia | unknown | A | W507 | Europe-11-22 | USSR | unknown | A |
| W218 | Oceania-1-27 | Australia | unknown | A | W508 | Europe-11-23 | USSR | 816 | A |
| W219 | Africa-1-1 | Algeria | Mahon, Flaksberger 16167 | A | W509 | Europe-11-24 | USSR | unknown | A |
| W220 | Africa-1-2 | Algeria | Shatilov Exp Sta | A | W510 | Europe-11-25 | USSR | unknown | A |
| W221 | Africa-1-3 | Algeria | unknown | A | W511 | Europe-11-26 | USSR | unknown | A |
| W222 | Africa-1-4 | Algeria | unknown | A | W512 | Europe-11-27 | USSR | unknown | B |
| W223 | Africa-1-5 | Algeria | unknown | A | W513 | Europe-11-28 | USSR | unknown | A |
| W224 | Africa-1-6 | Algeria | Mahon | A | W514 | Europe-11-29 | USSR | unknown | A |
| W225 | Africa-2-1 | Canary Islands | unknown | A | W515 | Europe-11-30 | USSR | Tulun 458 | A |
| W226 | Africa-2-2 | Canary Islands | unknown | A | W516 | Europe-11-31 | USSR | Valki Exp Sta 4 | A |
| W227 | Africa-2-3 | Canary Islands | unknown | A | W517 | Europe-11-32 | USSR | unknown | A |
| W228 | Africa-2-4 | Canary Islands | unknown | A | W518 | Europe-11-33 | USSR | unknown | A |
| W229 | Africa-2-5 | Canary Islands | unknown | B | W519 | Europe-11-34 | USSR | unknown | A |
| W230 | Africa-2-6 | Canary Islands | unknown | B | W520 | Europe-11-35 | USSR | unknown | A |
| W231 | Africa-2-7 | Canary Islands | unknown | A | W521 | Europe-11-36 | USSR | Abi | A |
| W232 | Africa-2-8 | Canary Islands | unknown | A | W522 | Europe-11-37 | USSR | Dickson J G 294 | A |
| W233 | Africa-2-9 | Canary Islands | unknown | A | W523 | Europe-11-38 | USSR | unknown | A |
| W234 | Africa-2-10 | Canary Islands | unknown | A | W524 | Europe-11-39 | USSR | unknown | A |
| W235 | Africa-2-11 | Canary Islands | unknown | A | W525 | Europe-11-40 | USSR | unknown | A |
| W236 | Africa-2-12 | Canary Islands | unknown | B | W526 | Europe-11-41 | USSR | Odessa Exp Sta | A |
| W237 | Africa-2-13 | Canary Islands | unknown | A | W527 | Europe-11-42 | USSR | unknown | A |
| W238 | Africa-3-1 | Morocco | unknown | A | W528 | Europe-11-43 | USSR | unknown | A |
| W239 | Africa-3-2 | Morocco | unknown | A | W529 | Europe-11-44 | USSR | unknown | A |
| W240 | Africa-3-3 | Morocco | unknown | A | W530 | Europe-11-45 | USSR | Valki Exp Sta 1 | A |
| W241 | Africa-3-4 | Morocco | unknown | B | W531 | Europe-11-46 | USSR | unknown | A |
| W242 | Africa-3-5 | Morocco | Mahan | A | W532 | Europe-11-47 | USSR | unknown | A |
| W243 | Africa-3-6 | Morocco | unknown | A | W533 | Europe-11-48 | USSR | Dickson J G 302 | A |
| W244 | Africa-3-7 | Morocco | unknown | A | W534 | Europe-11-49 | USSR | unknown | B |
| W245 | Africa-3-8 | Morocco | unknown | A | W535 | Europe-11-50 | USSR | Valki Exp Sta 1 | A |
| W246 | Africa-3-9 | Morocco | unknown | A | W536 | Europe-11-51 | USSR | unknown | A |
| W247 | Africa-3-10 | Morocco | unknown | A | W537 | Europe-11-52 | USSR | unknown | A |
| W248 | Africa-3-11 | Morocco | unknown | A | W538 | Europe-11-53 | USSR | unknown | B |
| W249 | Africa-3-12 | Morocco | unknown | A | W539 | Europe-12-1 | Yugoslavia | unknown | A |
| W250 | Africa-3-13 | Morocco | unknown | A | W540 | Europe-12-2 | Yugoslavia | unknown | A |
| W251 | Africa-3-14 | Morocco | Mahan | A | W541 | Europe-12-3 | Yugoslavia | Rustic winter | A |
| W252 | Africa-3-15 | Morocco | unknown | A | W542 | Europe-12-4 | Yugoslavia | Bon Fermier | A |
| W253 | Africa-3-16 | Morocco | unknown | A | W543 | Europe-12-5 | Yugoslavia | Potiska | B |
| W254 | Africa-3-17 | Morocco | Trigo duros | A | W544 | Europe-12-6 | Yugoslavia | Bon Fermier | A |
| W255 | Africa-4-1 | Tunisia | Bartletta | A | W545 | Europe-12-7 | Yugoslavia | Native winter | B |
| W256 | Africa-4-2 | Tunisia | Sbei Noir | A | W546 | Europe-12-8 | Yugoslavia | Native winter | A |
| W257 | Africa-4-3 | Tunisia | Louri | A | W547 | Europe-12-9 | Yugoslavia | unknown | A |
| W258 | Africa-4-4 | Tunisia | Native hard | A | W548 | Europe-12-10 | Yugoslavia | unknown | A |
| W259 | Africa-4-5 | Tunisia | Sbei 292 | A | W549 | Europe-12-11 | Yugoslavia | Native winter | A |
| W260 | Africa-4-6 | Tunisia | Mahmoudi A.P.3. | A | W550 | Europe-12-12 | Yugoslavia | unknown | A |
| W261 | Africa-4-7 | Tunisia | Mahmoudi | A | W551 | Europe-12-13 | Yugoslavia | unknown | A |
| W262 | Africa-4-8 | Tunisia | Biskri | A | W552 | Europe-12-14 | Yugoslavia | unknown | A |
| W263 | Africa-4-9 | Tunisia | Biskri | B | W553 | Europe-12-15 | Yugoslavia | unknown | A |
| W264 | Africa-4-10 | Tunisia | Native hard | A | W554 | Europe-12-16 | Yugoslavia | unknown | A |
| W265 | Africa-4-11 | Tunisia | ferrugineum Al., Flaksberger 17525 | A | W555 | Europe-12-17 | Yugoslavia | unknown | A |
| W266 | Africa-4-12 | Tunisia | Mahmoudi | A | W556 | Europe-12-18 | Yugoslavia | unknown | A |
| W267 | Africa-4-13 | Tunisia | unknown | A | W557 | Europe-12-19 | Yugoslavia | unknown | A |
| W268 | Africa-4-14 | Tunisia | unknown | A | W558 | Europe-12-20 | Yugoslavia | Veliko Hoce | A |
| W269 | Europe-1-1 | Bulgaria | Bebrovo | B | W559 | Europe-12-21 | Yugoslavia | unknown | A |
| W270 | Europe-1-2 | Bulgaria | Constantin | A | W560 | Europe-12-22 | Yugoslavia | unknown | A |
| W271 | Europe-1-3 | Bulgaria | Bebrovo | A | W561 | Europe-12-23 | Yugoslavia | Veliko Hoce | A |
| W272 | Europe-1-4 | Bulgaria | unknown | A | W562 | Europe-12-24 | Yugoslavia | unknown | A |
| W273 | Europe-1-5 | Bulgaria | unknown | A | W563 | Europe-12-25 | Yugoslavia | Veliko Hoce | A |
| W274 | Europe-1-6 | Bulgaria | unknown | A | W564 | Europe-12-26 | Yugoslavia | Prolific | A |
| W275 | Europe-1-7 | Bulgaria | unknown | A | W565 | Europe-12-27 | Yugoslavia | Native winter | A |
| W276 | Europe-1-8 | Bulgaria | Golema Franga | A | W566 | Europe-12-28 | Yugoslavia | Winter No. 6 | A |
| W277 | Europe-1-9 | Bulgaria | Bregari | A | W567 | Europe-12-29 | Yugoslavia | Native winter | A |
| W278 | Europe-1-10 | Bulgaria | unknown | A | W568 | Europe-12-30 | Yugoslavia | Veliko Hoce | B |
| W279 | Europe-1-11 | Bulgaria | Shumen | A | W569 | Europe-12-31 | Yugoslavia | Native winter | A |
| W280 | Europe-1-12 | Bulgaria | unknown | A | W570 | Europe-12-32 | Yugoslavia | unknown | A |
| W281 | Europe-2-1 | Crete | Blé mavratheri | A | W571 | Europe-12-33 | Yugoslavia | unknown | A |
| W282 | Europe-2-2 | Crete | unknown | A | W572 | Europe-12-34 | Yugoslavia | unknown | A |
| W283 | Europe-2-3 | Crete | Leventis (Blé de Pologne) | B | W573 | Europe-12-35 | Yugoslavia | Winter No.10 | B |
| W284 | Europe-2-4 | Crete | unknown | A | W574 | Europe-12-36 | Yugoslavia | unknown | A |
| W285 | Europe-2-5 | Crete | Blé cheliostazo | A | W575 | Europe-12-37 | Yugoslavia | Potiska | A |
| W286 | Europe-2-6 | Crete | unknown | A | W576 | Europe-12-38 | Yugoslavia | Native winter | A |
| W287 | Europe-2-7 | Crete | unknown | A | W577 | Europe-12-39 | Yugoslavia | Rustic winter | A |
| W288 | Europe-2-8 | Crete | unknown | A | W578 | Europe-12-40 | Yugoslavia | Rustic winter | A |
| W289 | Europe-2-9 | Crete | unknown | A | W579 | Europe-12-41 | Yugoslavia | unknown | A |
| W290 | Europe-3-1 | France | Alliés | A | W580 | Europe-12-42 | Yugoslavia | unknown | A |

“A” represents the allele *GS34-7Ba*; “B” represents the allele *GS34-7Bb*.

**Table S12.** Paralogous (*Ta-Ta*) and orthologous (*Ta-Os* and *Ta-At*) *GASR* gene pairs.

| *Ta-Ta* | *Ta-Os* | *Ta-At* |
| --- | --- | --- |
| *TaGASR1/TaGASR5* | *TaGASR1/OsGASR6* | *TaGASR21/AtGASR15* |
| *TaGASR1/TaGASR6* | *TaGASR3/OsGASR11* | *TaGASR25/AtGASR15* |
| *TaGASR2/TaGASR3* | *TaGASR5/OsGASR6* | *TaGASR27/AtGASR7* |
| *TaGASR2/TaGASR4* | *TaGASR6/OsGASR6* | *TaGASR28/AtGASR15* |
| *TaGASR2/TaGASR7* | *TaGASR10/OsGASR4* | *TaGASR33/AtGASR9* |
| *TaGASR3/TaGASR7* | *TaGASR13/OsGASR9* | *TaGASR33/AtGASR10* |
| *TaGASR4/TaGASR7* | *TaGASR16/OsGASR9* | *TaGASR34/AtGASR9* |
| *TaGASR5/TaGASR6* | *TaGASR17/OsGASR4* | *TaGASR34/AtGASR10* |
| *TaGASR8/TaGASR15* | *TaGASR18/OsGASR2* | *TaGASR34/AtGASR14* |
| *TaGASR9/TaGASR10* | *TaGASR19/OsGASR2* | *TaGASR36/AtGASR7* |
| *TaGASR9/TaGASR14* | *TaGASR20/OsGASR10* | *TaGASR36/AtGASR9* |
| *TaGASR9/TaGASR17* | *TaGASR21/OsGASR3* | *TaGASR36/AtGASR10* |
| *TaGASR10/TaGASR14* | *TaGASR22/OsGASR3* | *TaGASR36/AtGASR14* |
| *TaGASR10/TaGASR17* | *TaGASR23/OsGASR3* |  |
| *TaGASR13/TaGASR16* | *TaGASR24/OsGASR10* |  |
| *TaGASR14/TaGASR17* | *TaGASR25/OsGASR3* |  |
| *TaGASR18/TaGASR19* | *TaGASR26/OsGASR3* |  |
| *TaGASR20/TaGASR24* | *TaGASR27/OsGASR10* |  |
| *TaGASR20/TaGASR27* | *TaGASR28/OsGASR3* |  |
| *TaGASR21/TaGASR22* | *TaGASR29/OsGASR3* |  |
| *TaGASR21/TaGASR25* | *TaGASR33/OsGASR7* |  |
| *TaGASR21/TaGASR28* | *TaGASR33/OsGASR10* |  |
| *TaGASR22/TaGASR23* | *TaGASR34/OsGASR7* |  |
| *TaGASR22/TaGASR25* | *TaGASR34/OsGASR10* |  |
| *TaGASR22/TaGASR26* | *TaGASR36/OsGSR1* |  |
| *TaGASR22/TaGASR28* | *TaGASR36/OsGASR10* |  |
| *TaGASR22/TaGASR29* | *TaGASR37/OsGASR8* |  |
| *TaGASR23/TaGASR25* |  |  |
| *TaGASR23/TaGASR26* |  |  |
| *TaGASR23/TaGASR28* |  |  |
| *TaGASR23/TaGASR29* |  |  |
| *TaGASR24/TaGASR27* |  |  |
| *TaGASR25/TaGASR26* |  |  |
| *TaGASR25/TaGASR28* |  |  |
| *TaGASR25/TaGASR29* |  |  |
| *TaGASR26/TaGASR28* |  |  |
| *TaGASR26/TaGASR29* |  |  |
| *TaGASR28/TaGASR29* |  |  |
| *TaGASR33/TaGASR36* |  |  |
| *TaGASR34/TaGASR36* |  |  |

Note: *Ta*: *Triticum aestivum*; *Os*: *Oryza sativa*

Table S13 Names of 186 wheat varieties included in the natural population (NP)

| **Number** | **Variety** | **Number** | **Variety** | **Number** | **Variety** |
| --- | --- | --- | --- | --- | --- |
| 1 | Yangmai158 | 63 | Xinmai19 | 125 | Xinmai23 |
| 2 | Annong9267 | 64 | Linyou8159 | 126 | Yangmai19 |
| 3 | Annong92484W | 65 | Yangmai20 | 127 | Xingmai6 |
| 4 | Wanmai38 | 66 | Taikong6 | 128 | Yumai7 |
| 5 | Aizao64xi | 67 | Yangnuomai1 | 129 | Qin079984-14 |
| 6 | Zheng9023 | 68 | Neimai8 | 130 | Xiannong1 |
| 7 | Bainong64 | 69 | Neimai836 | 131 | Junmai35 |
| 8 | Xiaoyan6hao | 70 | Qinmai18 | 132 | Mingtian0417 |
| 9 | Zhoumai16 | 71 | Changhe25 | 133 | Womai8 |
| 10 | 02P67 | 72 | Yanzhan4110 | 134 | Xinong622 |
| 11 | Handan6172 | 73 | Fengdecunmai5 | 135 | Huapei8 |
| 12 | Aikang58 | 74 | Zhengyumai518 | 136 | Fanmai8 |
| 13 | Zhoumai18 | 75 | Luyuan502 | 137 | Huaimai05155(Huaimai30) |
| 14 | Y14 | 76 | Annong1124 | 138 | Yannong24 |
| 15 | Zhengmai3666 | 77 | Lunxuan988 | 139 | Yan2415 |
| 16 | X9610 | 78 | Bainong207 | 140 | Sumai3 |
| 17 | Zhoumai22 | 79 | Zhongmai895 | 141 | Jinmai60 |
| 18 | Xinmai18 | 80 | Zhoumai27 | 142 | Jingdong10 |
| 19 | Xinong889 | 81 | Anong0942-13 | 143 | Shixin733 |
| 20 | Huaihe0308 | 82 | Hengguan35 | 144 | Jimai34 |
| 21 | Wan52 | 83 | Su553 | 145 | Henong326 |
| 22 | Huaimai0320 | 84 | Yannong21 | 146 | Jifeng703 |
| 23 | Jimai17 | 85 | Zhongluotiegan | 147 | Jinmai31 |
| 24 | Jimai20 | 86 | Yang18 | 148 | Hengyou18 |
| 25 | Jimai22 | 87 | Annong1243 | 149 | Cangmai119 |
| 26 | 876 | 88 | Annong1274 | 150 | Gaoyou9415 |
| 27 | Mianmai39 | 89 | Yannong19 | 151 | Shimai16 |
| 28 | Shijiazhuang8 | 90 | Zhoumai28 | 152 | Baiyingmai2 |
| 29 | Shijiazhuang15 | 91 | Luo3429 | 153 | Helongkangbai4 |
| 30 | Lankao298 | 92 | Huarui0049 | 154 | Baomai8 |
| 31 | Ligao6 | 93 | Xumai9074 | 155 | Shimai15 |
| 32 | Fanmai5 | 94 | Huaimai0882 | 156 | Baomai3 |
| 33 | Kaimai18 | 95 | Luo6073 | 157 | Zhengmai98 |
| 34 | Annong0822 | 96 | Weilai0818 | 158 | Zhongmai155 |
| 35 | Ni1vt14 | 97 | Xuke129 | 159 | Shanmai15 |
| 36 | Jinhe9123 | 98 | Zhongyanmai1 | 160 | Zheng004 |
| 37 | Guinong775 | 99 | Annong1241 | 161 | Yumai2 |
| 38 | 1RZ | 100 | Zhen08066 | 162 | Lumai23 |
| 39 | R146 | 101 | Ningfeng518 | 163 | Taishan23 |
| 40 | Yi00-119 | 102 | Yang08-4 | 164 | Linmai2 |
| 41 | Jinmai73 | 103 | 1g303 | 165 | Wennong6 |
| 42 | Chuanmai42 | 104 | 1g251 | 166 | Zhoumai19 |
| 43 | CP01-39-3-2-4 | 105 | Bainong419 | 167 | Xinong979 |
| 44 | Shimai12 | 106 | Glenlen | 168 | Zhoumai17 |
| 45 | Shimai18 | 107 | 02Y151 | 169 | Zhoumai11(Yumai51) |
| 46 | Jishi02-1 | 108 | E158 | 170 | Zhoumai23 |
| 47 | Bainongaikang58 | 109 | 03G7 | 171 | Jinnong4 |
| 48 | Anong1007 | 110 | Neixiang203 | 172 | Shimai14 |
| 49 | Langxing66 | 111 | Xinmai19023(Xinmai21) | 173 | Jing411 |
| 50 | Annong8455 | 112 | Jimai19 | 174 | Hongmangchun21 |
| 51 | Liangxing99 | 113 | ENESCO | 175 | Langzhongbaimaizi |
| 52 | Xuke1 | 114 | F1RO | 176 | Fulingxuxumai |
| 53 | 984121 | 115 | Tai10604 | 177 | Zitongnvermai |
| 54 | Henong825 | 116 | Mianmai37 | 178 | Suiningtuotuomai |
| 55 | Zhengmai883 | 117 | CP02-8-5-6-1 | 179 | Wangshuibai |
| 56 | Longke0901 | 118 | Shiyou17 | 180 | Baihuomai |
| 57 | Bainong898 | 119 | Ji5265 | 181 | Huangguaxian |
| 58 | R77/6 (pm21) | 120 | Zhoumai25 | 182 | Chadinghongmai |
| 59 | Yangmai16 | 121 | Hongwan3 | 183 | Tuhulutou |
| 60 | Annong0950 | 122 | Wankenmai081 | 184 | Hulutou |
| 61 | Tai18 | 123 | Dinghong208 | 185 | Baitutou |
| 62 | Yunong69 | 124 | Bainong3271(pm13) | 186 | Baiyuhua |

Table S14 Mann-Whitney U test results for thousand grain weight, grain length and width of *TaGASR34*

| Trait | Mann-Whitney U Statistic |
| --- | --- |
| 16-TGW | 0.428 |
| 17-TGW | 1.055 |
| 18-TGW | 0.294 |
| 16-GL | -0.984 |
| 17-GL | 0.224 |
| 18-GL | 0.306 |
| 16-GW | 0.578 |
| 17-GW | 0.285 |
| 18-GW | 1.19 |

Note: TGW denotes thousand grain weight; GL denotes Grain length; GW denotes Grain width.
