# Supplementary figures and images for: Identification and Analysis of the GASR Gene Family in Common Wheat (Triticum aestivum L.) and Characterization of TaGASR34, a Gene Associated With Seed Dormancy and Germination
Source: Front Genet. 2019 Oct 18;10:980. doi: 10.3389/fgene.2019.00980 (PMC6813915; doi:10.3389/fgene.2019.00980)

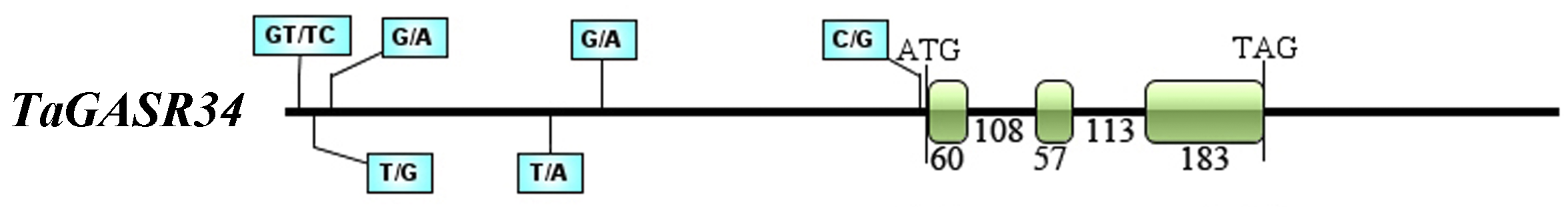

Supplement: Supplementary file 2 [file Image_1.tif]

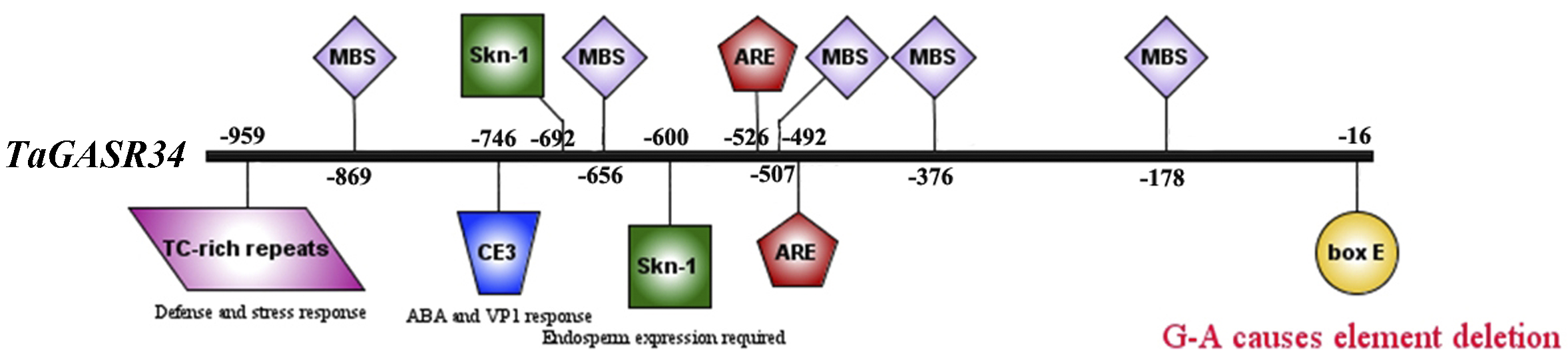

Supplement: Supplementary file 3 [file Image_2.tif]

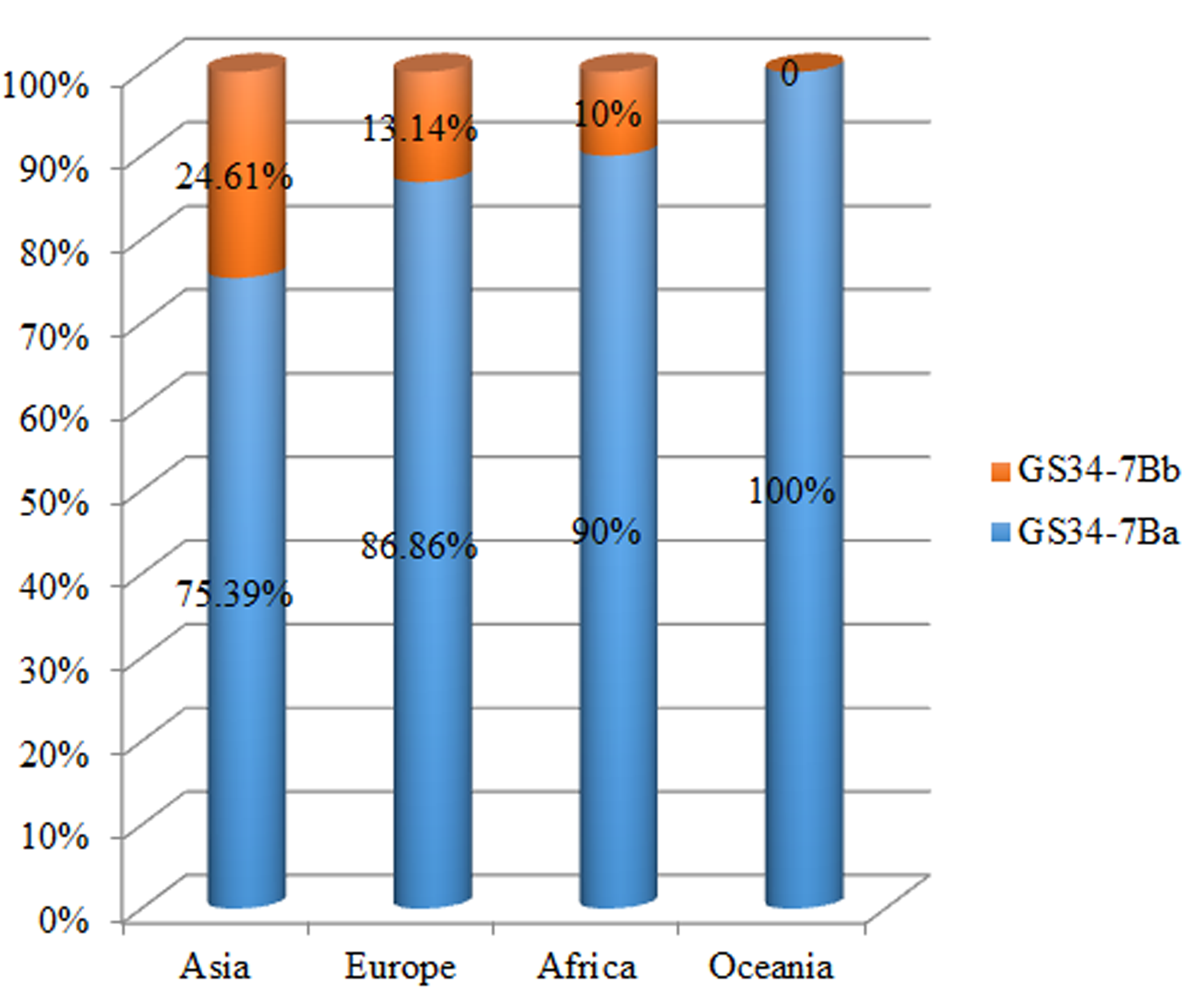

Supplement: Supplementary file 4 [file Image_3.tif]
